# A high-throughput effector screen identifies a novel small molecule scaffold for inhibition of ten-eleven translocation dioxygenase 2†

**DOI:** 10.1039/d2md00186a

**Published:** 2022-09-02

**Authors:** Shubhendu Palei, Jörn Weisner, Melina Vogt, Rajesh Gontla, Benjamin Buchmuller, Christiane Ehrt, Tobias Grabe, Silke Kleinbölting, Matthias Müller, Guido H. Clever, Daniel Rauh, Daniel Summerer

**Affiliations:** Department of Chemistry and Chemical Biology, TU Dortmund University and, Drug Discovery Hub Dortmund (DDHD), Zentrum für Integrierte Wirkstoffforschung (ZIW) Otto-Hahn Str. 4a 44227 Dortmund Germany guido.clever@tu-dortmund.de daniel.rauh@tu-dortmund.de daniel.summerer@tu-dortmund.de

## Abstract

Ten-eleven translocation dioxygenases (TETs) are the erasers of 5-methylcytosine (mC), the central epigenetic regulator of mammalian DNA. TETs convert mC to three oxidized derivatives with unique physicochemical properties and inherent regulatory potential, and it initializes active demethylation by the base excision repair pathway. Potent small molecule inhibitors would be useful tools to study TET functions by conditional control. To facilitate the discovery of such tools, we here report a high-throughput screening pipeline and its application to screen and validate 31.5k compounds for inhibition of TET2. Using a homogenous fluorescence assay, we discover a novel quinoline-based scaffold that we further validate with an orthogonal semi-high throughput MALDI-MS assay for direct monitoring of substrate turnover. Structure–activity relationship (SAR) studies involving >20 derivatives of this scaffold led to the identification of optimized inhibitors, and together with computational studies suggested a plausible model for its mode of action.

## Abstract 2

TET dioxygenases are the erasers of 5-methylcytosine in mammalian DNA. We report a high-throughput screen and identify a low micromolar TET2 inhibitor. Computational studies suggest that the inhibitor uses a novel competitive binding mode.

## Graphical abstract

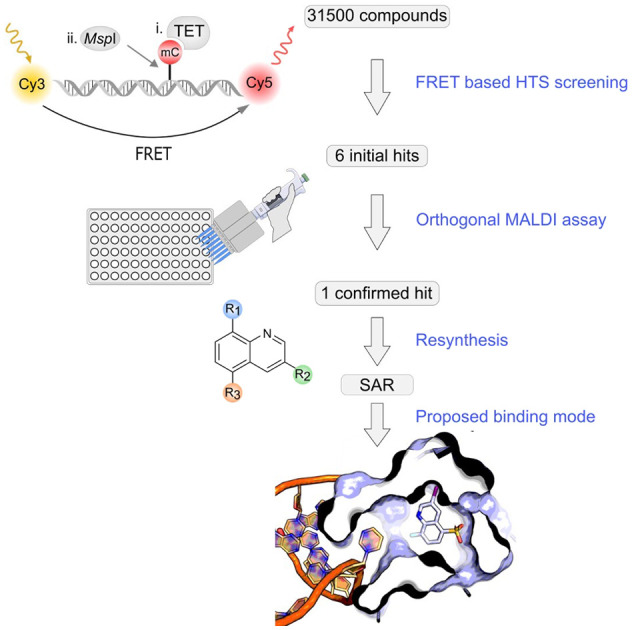

## Introduction

**Scheme 1:**
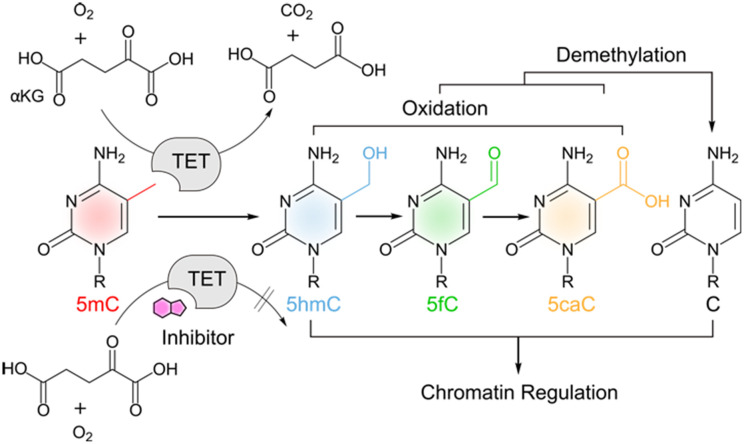
TET oxidation of 5-methylcytosine (5mC) and downstream events.

TET proteins are alpha-ketoglutarate (α-KG), Fe(ii) and oxygen-dependent dioxygenases that catalyse the iterative oxidation of 5-methylcytosine (mC) to 5-hydroxymethylcytosine (hmC), 5-formylcytosine (fC) and 5-carboxylcytosine (caC) in mammalian DNA (Scheme 1).^1–4^ This activity is not only integral to the active demethylation of DNA, but also creates a complex landscape of oxidized mC derivatives that can uniquely interact with chromatin-associated proteins^5–8^ with potential functions for the regulation of gene expression.^9,10^ TETs have been shown to play a vital role in early embryonic development and in the onset of diseases including cancer.^11,12^ There is a growing interest in the conditional control of TET activity by molecular tools such as engineered TETs^13,14^ and inhibitors based on cyclic peptides^15^ or small molecules.^16–19^ The latter are particularly useful candidates for functional control of TET activity in complex biological environments. Moreover, properly validated and characterized small molecules to perturb and modulate target enzyme functions are the groundwork for developing successful candidates for clinical use.^20^

Recent efforts have afforded the first small molecule inhibitors of TETs^17–19^ (the cytosine-based compound Bobcat339 (ref. 21) has recently been shown to not be an inhibitor of TET1/2).^22^ However, a particular challenge of inhibiting TETs is their dependence on abundant substrates and cofactors that have additional key functions in cells and are used by a large number of related enzymes. This complicates the use of substrate analogues with high structural similarity, or the use of compounds that alter cofactor availability. For example, *N*-oxalylglycine (NOG) and its esterified derivative dimethyloxalylglycine (DMOG)^16^ are popular TET inhibitors that need to be applied in millimolar concentrations and differ only minimally in structure from α-KG, an abundant metabolite with numerous functions in cell physiology.^23^ Other inhibitors rely on the complexation of free Fe(ii) (*e.g.* deferoxamine, DFOA^24^). Hence, there is a need to identify novel scaffolds that differ more substantially from the natural substrates and thus provide new avenues for the design of selective inhibitors. For example, an inhibitor was recently identified by a pure *in silico* screening that was proposed to bind the TET active site without competing with α-KG.^17^

To enable an effective experimental access to the discovery of novel small molecule TET inhibitors, we here report a high throughput pipeline for the screening and validation of TET inhibitors from large compound collections. We developed a homogenous one-pot FRET-assay for quantification of TET activity *in vitro* and employed it for screening a compound library comprising 31.5k chemically diverse molecules. We identified an inhibitor of human TET2 based on a novel tri-substituted, sulfonamide-derivatized quinoline scaffold. Careful hit validation and structure–activity relationships using a derivative library of this scaffold with an orthogonal MALDI-MS assay revealed a simple sulfonic acid derivative as low micromolar inhibitor of TET2 and together with computational studies provided clues to its mode of action.

## Results

### Development of a robust high-throughput screening assay

To develop an HTS pipeline and identify TET inhibitors, we chose TET2 as a target because of its key role in the development of hematopoietic malignancies.^11^ A central requirement for our goal was a robust screening assay for measuring TET2 activity in a highly parallel fashion. Although oxidized mC nucleobase products (*e.g.* hmC) in DNA can be detected *via* antibodies, enzymatic assays or mass spectrometry,^25–27^ there had been no one-pot homogenous fluorescence assays available with high potential for actual high-throughput settings.

**Fig. 1:**
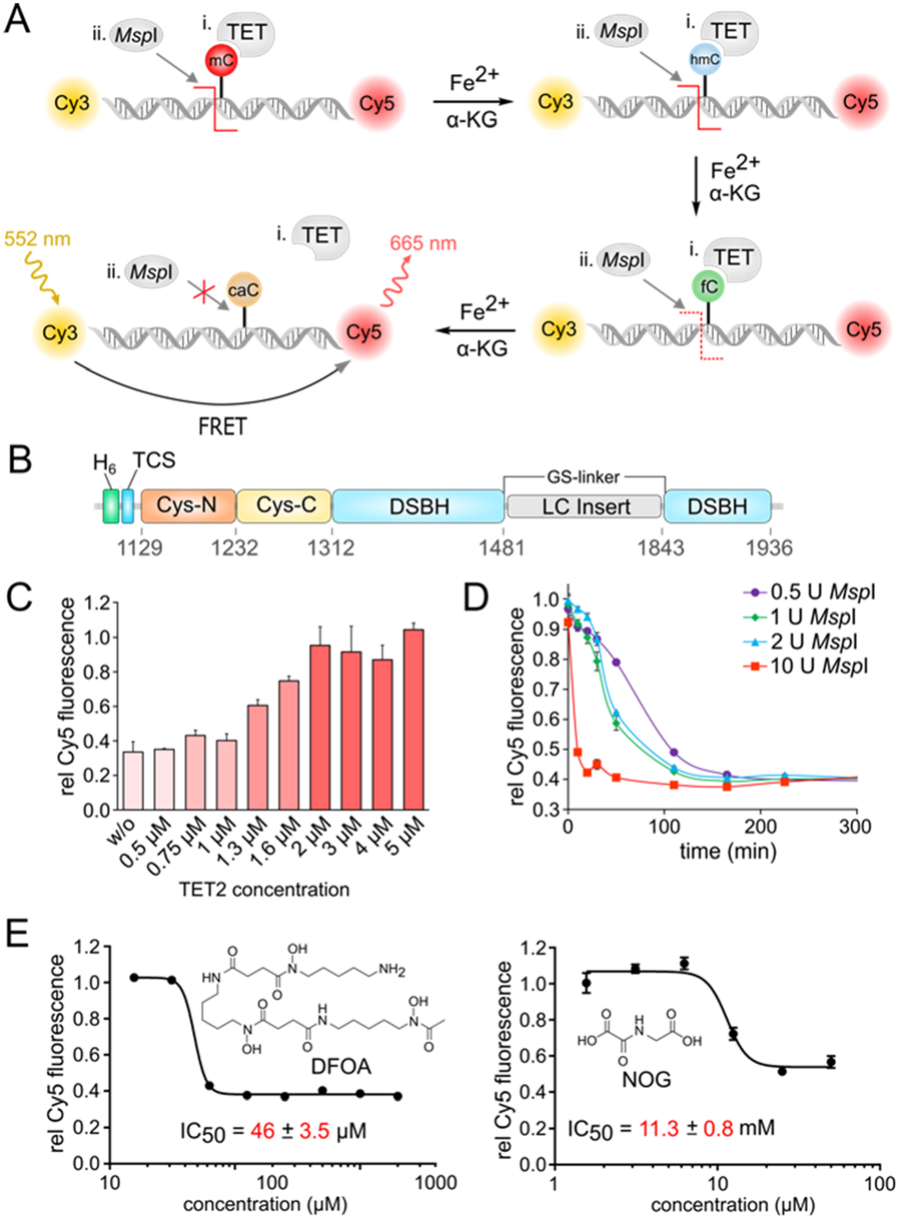
Homogeneous fluorescence assay for HT screening of TET inhibitors. A) Overview of the *Msp*I-based FRET assay for measuring TET2 activity in presence of O_2_*in vitro*. Forward oligo: 5′-GGGAC^m^CGGAGGG-3′, reverse oligo: 5′-CCCTCCGGTCCC-3′. B) Domain structure of TET2 catalytic domain (TET2-CD) used in the study; Cys-N/C = N/C-terminal cysteine rich domain, TCS = thrombin cleavage site, LC insert = low complexity insert, DBSH = double stranded β-helix. C) Determination of TET2 assay concentration for quantitative mC oxidation and inhibition of *Msp*I digestion at 20 U. Error bars show standard deviation from four replicate experiments. D) Determination of minimal *Msp*I concentration for quantitative digest of oligo duplex at 0.5 μM under HT assay conditions. Error bars show standard deviation from two replicate experiments. E) Dose response and IC_50_ determination of broad-spectrum dioxygenase inhibitors DFOA and NOG with the FRET assay. Error bars showing the standard deviation from two technical replicate experiments. IC_50_ values are obtained from three independent experiments with technical duplicates each.

We first developed an assay based on the ability of the methylation-sensitive restriction endonuclease *Msp*I to cleave target DNA duplexes containing a CpG dinucleotide with an mC, hmC, or fC, but not caC in its recognition motif.^28^ Use of a DNA duplex terminally labelled with a FRET donor/acceptor dye pair (Cy3/Cy5) thereby enables measuring the amount of caC product generated from an mC duplex by TET (Fig. 1A). We expressed and purified the catalytic domain of TET2 (TET2-CD) with an N-terminal His_6_-tag followed by a thrombin cleavage site (Fig. 1B and S1†). We replaced the low-complexity (LC) insert of the catalytic domain by a GS linker for better expression in *E. coli*, since this has been shown to not affect its activity *in vitro*.^29^ We then designed a 12-bp oligonucleotide containing a central *Msp*I recognition sequence (‘CCGG’) containing an mC nucleobase in the CpG dinucleotide, and hybridized it with the Cy3/Cy5-labeled antisense strand. We identified an universal buffer system (50 mM potassium acetate, 20 mM Tris-acetate, 10 mM magnesium acetate, 100 μg ml^−1^ BSA, pH 7.9) that was compatible with the activity of both TET2 and *Msp*I, and thus was applicable to one pot reactions without intermediate purification steps. We incubated 0.5 μM of the DNA duplex with TET2 at different concentrations under aerobic conditions and in the presence of essential assay components, *i.e.* α-KG, ascorbate, dithiothreitol (DTT), and Fe(ii) at 37 °C and pH 8 for 1 hour. We then added 20 U *Msp*I enzyme to determine the caC level generated by TET2-mediated oxidation. We observed a high Cy5 fluorescence (high FRET) only for reaction mixtures containing TET2, indicating successful oxidation of mC and blockage of *Msp*I. A 4-fold molar excess of TET2 enzyme (2 μM) was thereby sufficient for a quantitative oxidation of mC in 1 hour at 37 °C and subsequent inhibition of *Msp*I activity (Fig. 1C).

For an assay upscale, we chose a concentration of 1 U *Msp*I per reaction to achieve quantitative cleavage and baseline Cy5 fluorescence after incubation at RT for 4 hours (Fig. 1D, see Fig. S2† for optimization of duplex concentration).

As a final requirement for HTS, we found that the assay can readily tolerate up to 4% final DMSO concentration without altering TET2 or *Msp*I enzymatic activity (Fig. S3†), making it highly compatible with the screening of compound collections stored in standard DMSO stocks. The *Z*-factor (*Z*′) of this optimized FRET assay in 384-well plates was 0.69, indicating a high robustness and compatibility with our screening conditions (Fig. S4†).^30^

We then evaluated the ability of our screening assay to detect and profile the inhibitory activity of previously reported broad-spectrum dioxygenase inhibitors with two different modes of action. We thereby obtained for the Fe(ii) chelator DFOA and the α-KG analog NOG^16^ IC_50_ values of 46 μM and 11.3 mM, respectively (Fig. 1E).

### High-throughput screening leads to identification of potent hits

**Fig. 2:**
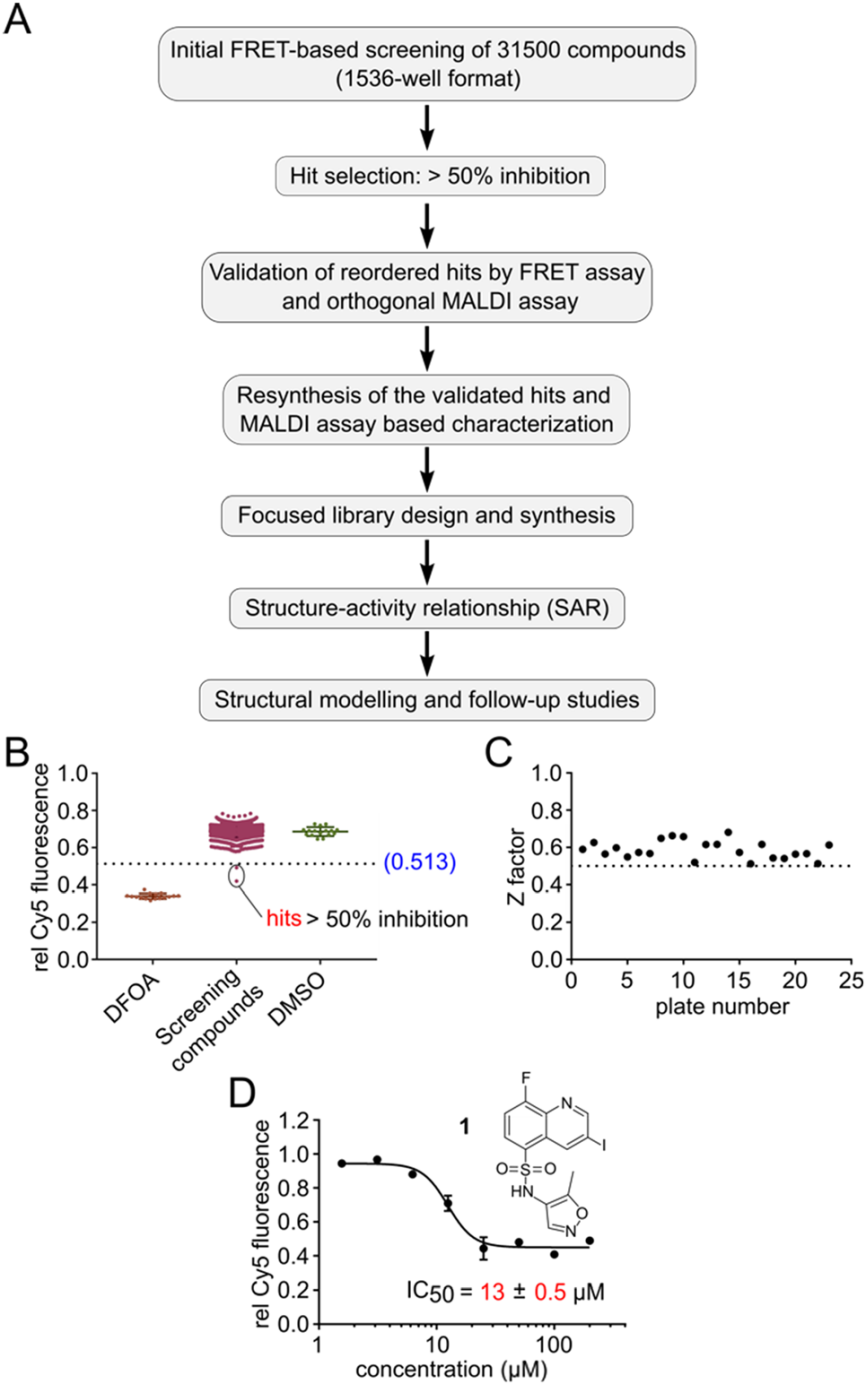
High-throughput screening pipeline for the discovery of TET2 inhibitors. A) Workflow of the pipeline. B) Representative FRET screening results from one 1536 well plate. DFOA = deferoxamine (positive control), DMSO as negative control. C) Global assay quality of all of the screening plates represented by *Z*-factor. D) Validation by dose response of hit 1 with the FRET assay. Note that the figure shows the hypothetical structure of 1 as indicated by the vendor. Error bars showing the standard deviation from two technical replicate experiments. IC_50_ values are obtained from three independent experiments with technical duplicates each.

With this HTS assay in hand we set out to screen our in-house compound library of 31.5k compounds in 1536-well plates. This chemically diverse library was designed to cover a broad chemical space with defined ranges of molecular weight as well as number of stereocenters and aromatic rings, with PAIN and Lipinski rule of 5 filters applied.^31^ We developed a pipeline for screening, selection, validation and resynthesis of the primary hit compounds, followed by a focused library design of the validated hits to study structure–activity relationship (SAR) (Fig. 2A). All experimental observations were further supported by structural modelling studies. Screening was performed utilizing the Robotics Assisted Screening Platform for Efficient Ligand Discovery (RASPELD) unit, which allows semi-automated screening in high throughput.^31^

For screening, we premixed the annealed oligonucleotides with TET2 and all other essential components except Fe(ii) in 1536-well plates, followed by the acoustic transfer of test compounds into each well at a final concentration of 50 μM. We preincubated the compounds with the mixture of oligonucleotide and TET2 enzyme for 30 minutes at RT in a humidified chamber before the Fe(ii) solution was added to start the reaction. We incubated the plate at 37 °C for 1 hour for TET2-mediated mC oxidation. Afterwards, we added *Msp*I and incubated the plates for an additional 4 hours at RT. In each screening plate, we included DMSO as negative (0% inhibition) and 1 mM DFOA as positive (100% inhibition) controls to determine the assay quality (*Z*′). In order to distinguish the false-positive hits, we normalized the final Cy5 fluorescence of each well to its respective initial value immediately after *Msp*I addition. We selected compounds as primary hits for further evaluation if they exhibited >50% inhibition of TET2 activity (Fig. 2B shows results of an exemplary 1536-well plate).

**Fig. 3:**
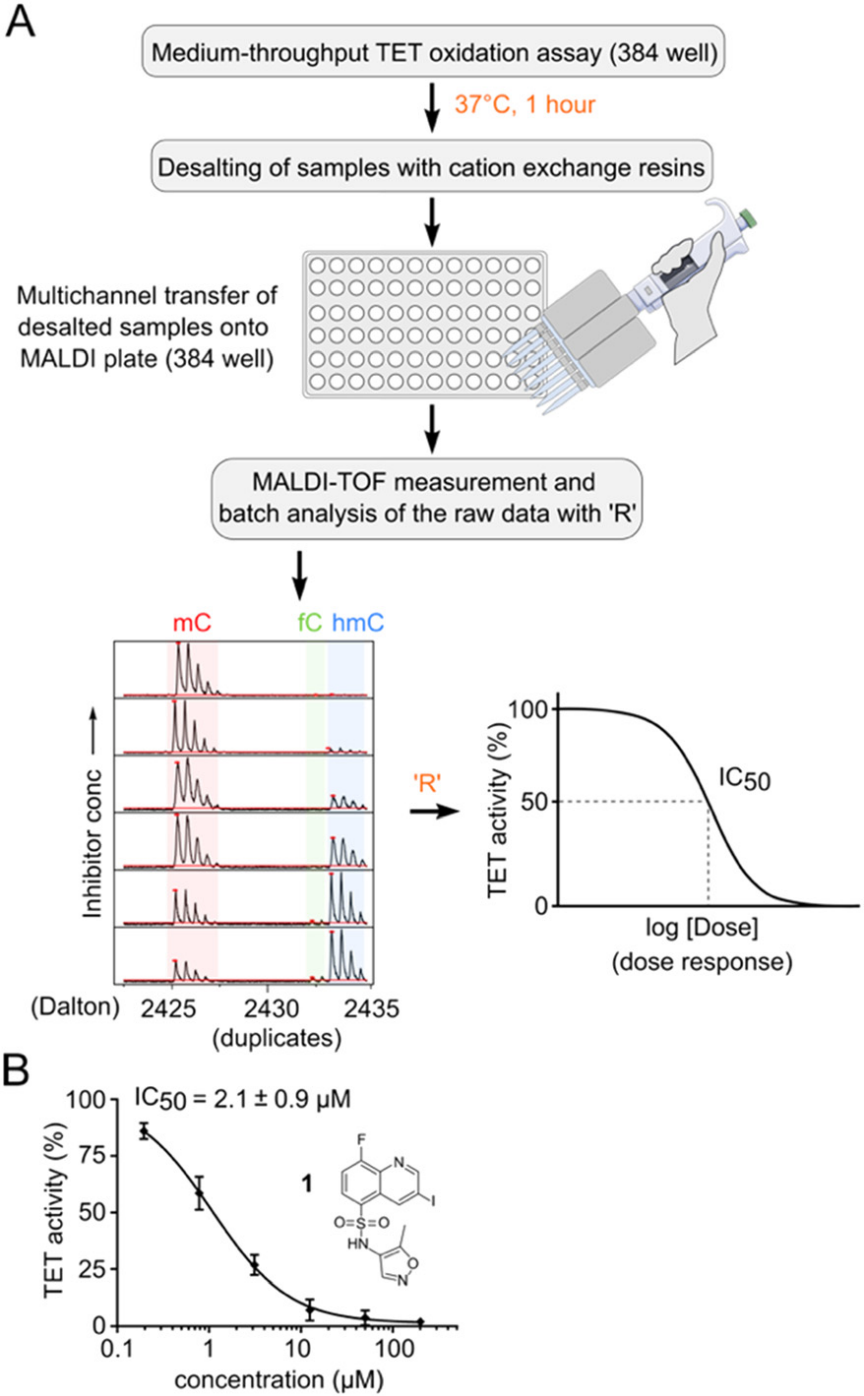
MALDI-MS assay and analysis pipeline for IC_50_ determination. A) Schematic representation of pipeline. B) Hit validation and dose response of compound 1 with an orthogonal MALDI assay. Note that the figure shows the hypothetical structure of 1 as indicated by the vendor. Error bars showing the standard deviation from two technical replicate experiments. IC_50_ values are obtained from three independent experiments with technical duplicates each.

In total, we screened 24 individual 1536-well plates yielding a global *Z*-factor of 0.59 ± 0.05, which confirmed the robustness of the FRET assay in a 1536-well screening format (Fig. 2C). We identified six primary hits (corresponding to a hit rate of 0.02%) for further evaluation. We reordered all six primary hit compounds freshly from commercial sources and performed dose-dependent analyses using the FRET assay in 384-well plates. We thereby identified compound 1, a quinoline scaffold linked to an isoxazole ring *via* a sulfonamide moiety to be the only active hit compound inhibiting TET2 in a dose-dependent manner (IC_50_ = 13 ± 0.5 μM) (Fig. 2D). The observed reduction of Cy5 fluorescence was not due to quenching by 1 (no reduction in Cy5 fluorescence intensity in the absence of *Msp*I enzyme was observed), indicating a specific inhibition of TET2 by 1 (Fig. S6†). Validation of hits with an orthogonal MALDI assay. In order to validate our primary screening results, we developed an orthogonal medium-throughput MALDI pipeline enabling the quantitative analysis of substrate turnover and formation of the TET2 products hmC and fC (Fig. 3A). Mass spectrometry-based methods have been previously employed to directly measure TET activity *in vitro*.^19,25,32^ We aimed to miniaturize such assays to adapt them to a plate-based, semi-HT assay setting. For this purpose, we used MALDI-TOF analysis and developed an ‘*R*’ pipeline for batch processing of the large amount of MALDI raw data. This allows automated quantification of the intensity of educt (mC) and products (hmC, fC) for rapid and accurate determination of IC_50_ values.

We used an annealed 8-mer palindromic oligonucleotide duplex (5′-CACmCGGTG-3′) as a substrate. We preincubated a mixture of 0.5 μM duplex and 1 μM TET2 along with the essential assay components with a serial dilution of the inhibitor in a humidified chamber at RT for 30 minutes. We then added Fe(ii) and incubated the mixture for one additional hour at 37 °C for TET2-catalyzed oxidation. For MALDI analysis, we desalted the reaction mixtures with cation exchange resin. We transferred the desalted samples to a 384-well MALDI sample plate in parallel using 3-hydroxypicolinic acid and diammonium hydrogen citrate as the matrix (Fig. 3A). We measured MALDI-TOF MS of the dose–response samples in duplicates, and batch-analyzed the raw data to determine absolute ion intensities of [M + H]^+^ ions of mC, hmC and fC (intensity of the caC ion was neglected due to its low abundance). We assumed that oxidation at a single CpG of the oligo would not change its ionization efficiency.^33^ We quantified the TET activity as the percentage of oxidized products (hmC + fC) formed, and then normalized that value to the negative DMSO control. We then revalidated our primary hit compound 1 using the orthogonal MALDI assay and determined the dose response curve with a corresponding IC_50_ value of 2.1 ± 0.9 μM (Fig. 3B and S7†). We used the MALDI assay for determination of all further dose response experiments and IC_50_ values because of its ability to directly quantify mC oxidation products.

### Structure–activity relationships reveal a novel tri-substituted quinoline scaffold for the inhibition of TET2 dioxygenase

We next resynthesized compound 1 to independently validate its inhibitory activity against TET2. To our surprise, we observed that the resynthesized compound 1 was inactive against TET2 at concentrations up to 200 μM (Table 1). In LC-MS analyses, we found minor impurities with masses corresponding to the cleavage of the sulfonamide bond as well as to defluorination in the DMSO stock solution (Fig. S5†).^34^ We thus reasoned that the resulting sulfonic acid 2 could be an active compound of the stock solution.

**Table 1:** Structure–activity relationship (SAR)

		IC_50_ (μm)			IC_50_ (μm)
1	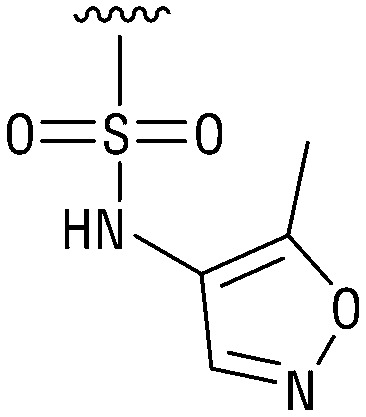	>200	10	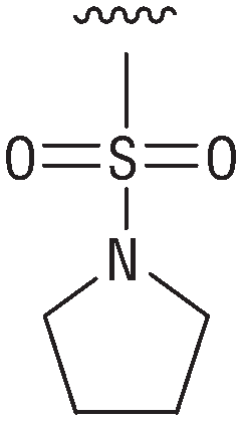	>200
2	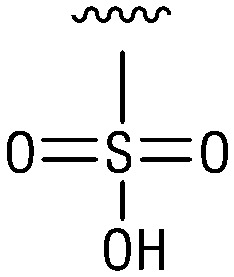	2.3 ± 0.6	11	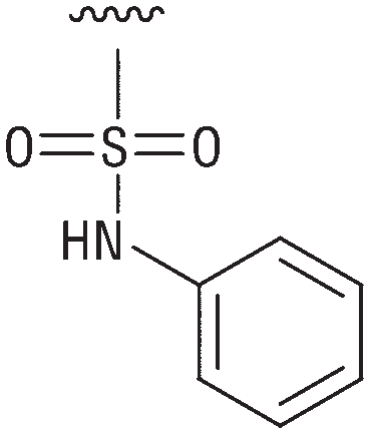	23.9 ± 4.1
3	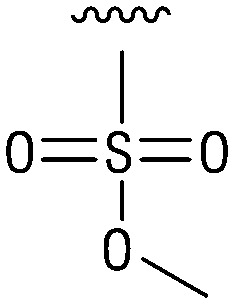	>200	12	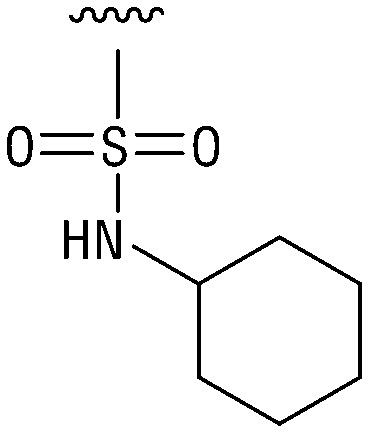	>200
4	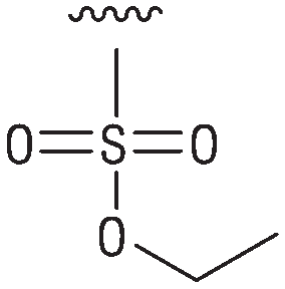	>200	13	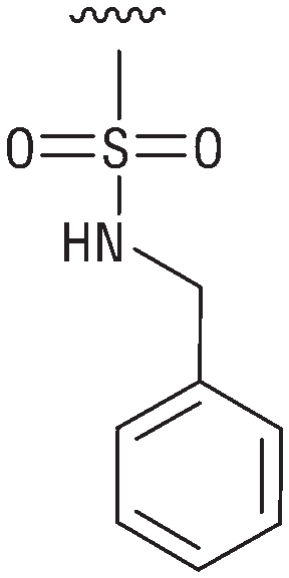	>200
5	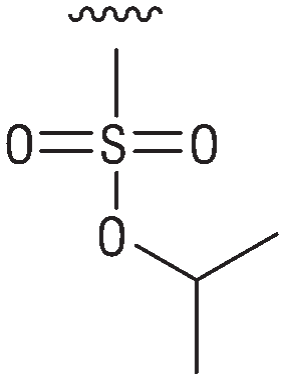	>200	14	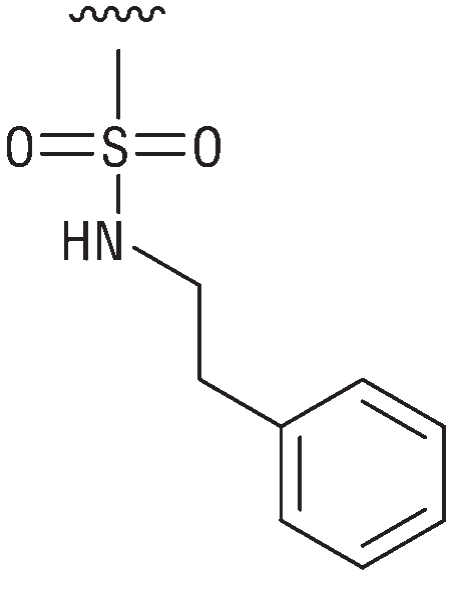	>200
6	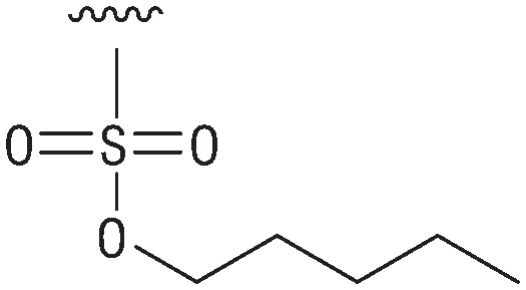	>200	15	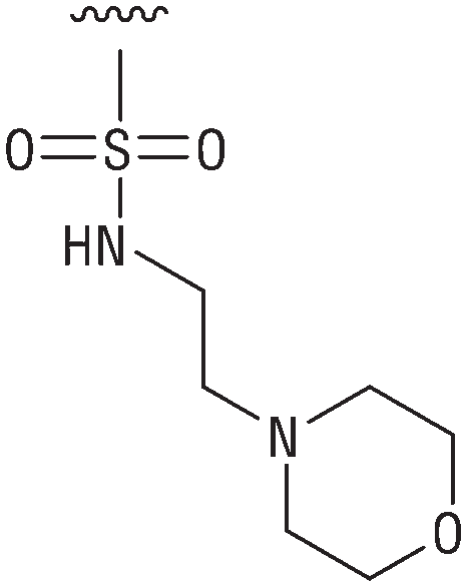	>200
7	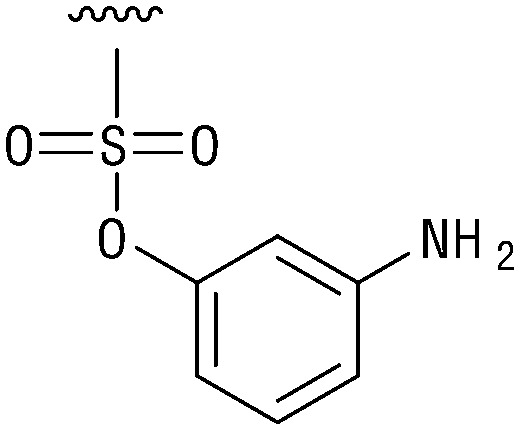	>200	16	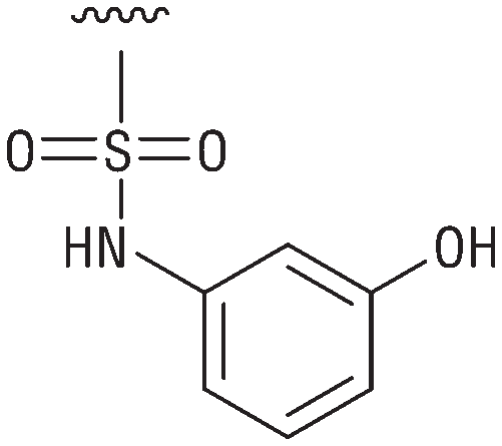	>200
8	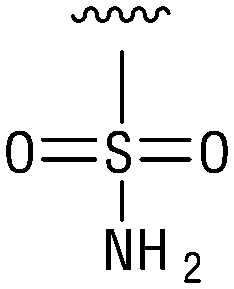	86.6 ± 5.6	17	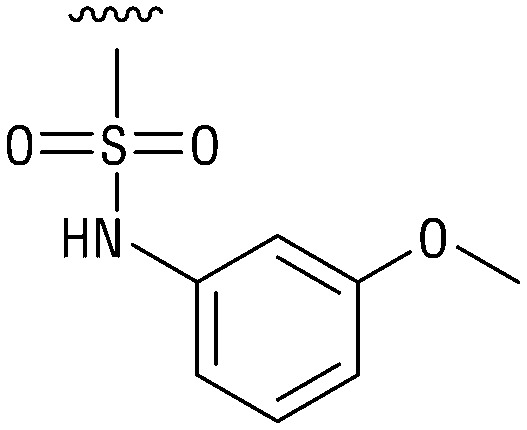	>200
9	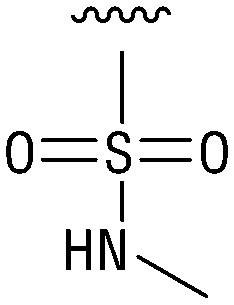	>200			

Indeed, resynthesized 2 showed a potent inhibition of TET2 activity, with an IC_50_ value of only 2.3 ± 0.6 μM (Table 1). 2 was thereby the only active sulfonic acid in our screen, suggesting that the specific scaffold of 2 was required for its activity (similarly, other sulfonic acids, such as *o*-anilinesulfonic acid, *p*-toluenesulfonic acid or 2-bromoisonicotinic acid showed no inhibition of TET2 in additional experiments, data not shown). The inactivity of 1 and the high activity of 2 indicated a key role of the substituent at the 5-position of the quinoline ring. We thus synthesized a focused library of derivatives with altered substituents at this position (and few derivatives with alterations at the fluorine and iodine position of 1; see Table 1). We first synthesized a series of sulfonic acid esters with gradually increasing steric demand of the alcohol moiety (3–7). Whereas these esters were all inactive, we observed that 3–6 showed activity after prolonged storage in DMSO, indicating hydrolysis to 2 (Fig. S10† shows data for 3). However, our library contained 27 additional sulfonic acid esters that were all inactive in our screen, further supporting the requirement of the specific scaffold of 2 for its activity. As a closely related compound to 2, we next tested the simple sulfonamide 8, and indeed observed activity (IC_50_ = 86.6 ± 5.6 μM). We were therefore interested, if other sulfonamides may also be active, and first tested the methyl-derivative 9 and the secondary pyrrolidine sulfonamide 10, which turned out to be inactive (Table 1). We however found that substitution with a phenyl ring resulted in even higher activity (IC_50_ of 23.9 ± 4.1 μM) as compared to the free sulfonamide 8 (Table 1). This activity required aromaticity of the ring, indicated by the inactivity of the cyclohexyl sulfonamide 12. Moreover, neither methylene nor ethylene bridges to the phenyl ring, nor further variations of the phenyl substituent were tolerated (13–17, Table 1). Similarly, a number of derivatives with alterations at the fluorine and iodine positions were inactive (Fig. S16†).

Hence, with a top-down systematic SAR approach we could streamline our initial screening results and identify 8-fluoro-3-iodoquinoline-5-sulfonic acid 2 as a novel scaffold that inhibits TET2 with a low μM IC_50_ value. This further highlights the importance of systematic resyntheses/validation and SAR for a successful discovery and development of potent inhibitors (see Fig. S8 and S9† for full inhibition profiles of the active compounds 2, 8 and 11).

### Molecular docking predicts a plausible binding mode

Next, we set out to investigate potential binding modes of 2 exploring two possible scenarios. In the first scenario, the inhibitor binds as an mC-competitor and prevents the essential base flipping step of TET2 catalysis that locks the substrate cytosine in the active site.^29^ To this end, we performed molecular docking studies with the software GOLD. First, we explored potential binding poses of the inhibitor in the nucleobase mC binding site. We found a unique binding pose throughout the highest-scored poses, which suggests ligand binding *via* hydrogen bonds with R1261 and the backbone nitrogen atom of R1262 (Fig. S11†). One of the two oxygen atoms of the sulfonic acid group thereby remains solvent-exposed, theoretically enabling the introduction of additional substituents as sulfonic acid esters.

However, ester derivatives of 2 (including the smallest methyl ester 3) were inactive in our MALDI assay (Table 1). Therefore, though this binding mode might interfere with DNA binding and base flipping, it is unlikely to be correct.

**Fig. 4:**
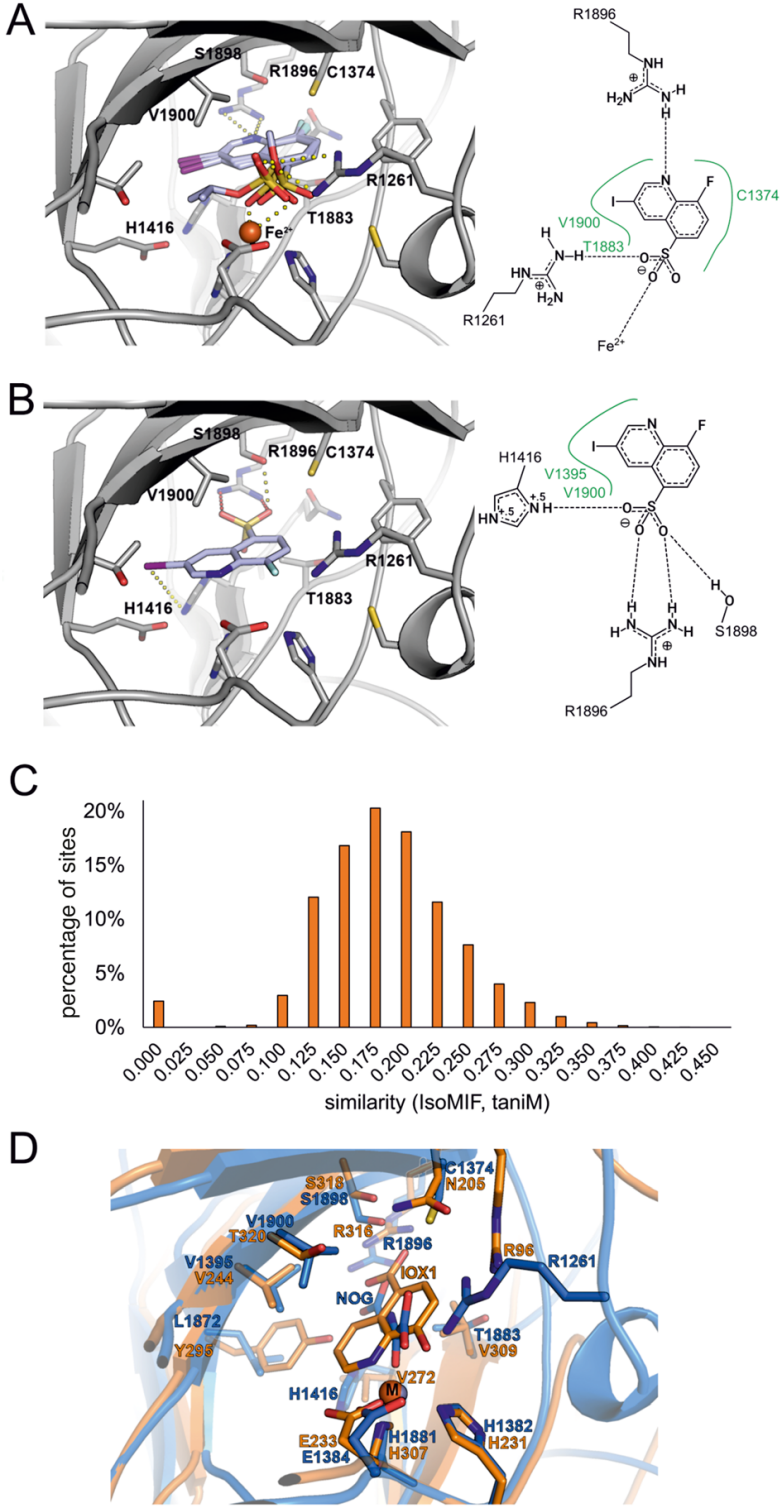
Binding hypothesis for 2. A) Compound 2 and its inactive ester derivates 3 and 5 were docked into the TET2 α-KG binding site in the presence of Fe(ii). The highest-scored binding pose of each compound is shown as light blue sticks. The protein is shown as grey cartoon with amino acid side chains within 4 Å of the docked compounds shown as grey sticks. The Fe(ii) ion is shown as orange sphere. The docking-derived binding modes do not explain the observed SAR for the compounds. A 2D-plot of potential residues interacting with active compound 2 in presence of Fe(ii) is shown on the right. B) Docking pose of active compound 2 docked in the absence of Fe(ii) ion depicted as in Fig. 4A. The docking pose can only be found for the active compound 2 as opposed to the esters 3 and 5 whose substituents would clash with side chains in proximity of R1896. C) Comparison of the TET2 α-KG site with IsoMIF. D) Structural alignment of fat mass and obesity associated protein FTO (PDB-ID: 4ie4) shown as orange cartoon with Fe(ii) as sphere (M) in complex with the α-KG analogue inhibitor 8-hydroxyquinoline-5-carboxylic acid (IOX1) and of TET2 (PDB-ID: 5deu) shown as blue cartoon in complex with Zn^2+^ (shown as sphere, M) and *N*-oxalylglycine (NOG). The structural similarity between 2 and IOX1 as well as the binding site similarity between the two dioxygenases corroborate the binding hypothesis of Fig. 4B. Figures were generated using UCSF Chimera.^35^

In the second scenario, the inhibitor binds in an α-KG-competitive manner. We thus analyzed potential binding modes in the α-KG binding pocket. To this end, we retained one water molecule and the metal ion (Fe(ii)) in the active site. Intriguingly, the resulting poses of 2 and the two inactive esters 3 and 5 serving as negative compounds showed a similar interaction profile in the binding site. The oxygen atoms of the sulfonic acid moieties of all three compounds were engaged in coordination to the Fe(ii) ion and formed hydrogen bonds to the R1261 side chain, whereas the quinoline nitrogen atoms formed hydrogen bonds with the side chain of R1896 (Fig. 4A). Though the deprotonated sulfonate of 2 is expected to coordinate Fe(ii) better than the uncharged esters of 3 and 5, this proposed binding mode does not explain the observed complete inactivity of 3 and 5.

We thus performed another docking study in that we aimed to avoid a potential score bias with respect to metal coordination by using a complex without the Fe(ii) ion as a working model. This time, we found a distinct binding mode of compound 2, which was significantly different from the binding mode of the two inactive esters. In this mode, the sulfonic acid group is engaged in a salt bridge with the R1896 side chain and in two additional hydrogen bonds to the side chains of H1416 and S1898 (Fig. 4B). The quinoline nitrogen atom is located at the position that was originally occupied by a coordinating water molecule (HOH2113, PDB-ID 5deu). The fluorine atom occupies the position of an oxygen atom of the coordinating carboxylic acid moiety of the α-KG cofactor. We did not find comparable binding modes for the inactive ester and amide derivatives of 2 (Table 1) due to a steric hindrance at the bottom of the α-KG pocket formed by R1896. Therefore, this binding mode – which is consistent with our SAR – is the mode that best explains the observed TET2 inhibition by 2 among the ones we obtained from our models.

### Binding site comparisons reveal similarities with the dioxygenase inhibitor IOX1, whereas DFT calculations suggest differences in chelating properties

To further test our binding hypothesis, we performed a binding site comparison analysis employing the α-KG binding pocket of the TET2-hmC complex structure (PDB-ID 5deu) as a template. We applied the binding site comparison tool IsoMIF^36^ to compare our target binding pocket to all druggable binding sites as stored in the scPDB,^37^ expecting to obtain very similar binding sites in complex with other ligands that could shed light on the potential binding mode of 2. IsoMIF has been previously applied in various medicinal chemistry projects and enabled the identification of similarities between binding sites of globally unrelated proteins.^38^ The similarity score ranges between 1 (identical) and 0 (completely dissimilar). For the comparison of our target binding site, we used the score taniM as a similarity measure, since it showed the most promising performance in a benchmark study.^39^ In our comparison to the TET2 binding site, this similarity score ranged between 0.1 and 0.3 for most binding site pairs (Fig. 4C). However, some of the binding sites in the scPDB showed similarity scores above 0.375. The most interesting similar binding sites were those of the two human dioxygenases hypoxia-inducible factor 1-alpha inhibitor (HIF1α, PDB-ID 3od4) and 2-oxoglutarate and iron-dependent oxygenase domain-containing protein 1 (OGFOD1, PDB-ID 4nhy). We used the resulting binding site alignments to compare all binding sites of both proteins to the α-KG binding site of TET2. A superposition that especially attracted our attention was the alignment with the structure of HIF1α (PDB-ID 3od4). Its binding site was crystallized with the compound IOX1 (8-hydroxyquinoline-5-carboxylic acid),^40^ that is based on the well-established chelator 8-hydroxyquinoline (8HQ). 8HQ is known to complex Fe(ii) and various other metal cations, and has broad biological activities by itself and in derivatized form, ranging from antimicrobial to anticancer to antifungal properties. IOX1 itself also has broad activity against oxygenases and was found in complex with other α-KG dependent enzymes in the PDB, such as fat mass and obesity associated protein FTO, AlkB, and JMJD2a.^40^ The differences of IOX1 to our hit compound 2 are the exchange of the hydroxyl moiety by the fluorine atom, the carboxylic acid by the sulfonic acid group and an additional iodine atom at the 3-position of the quinoline scaffold (Fig. 4D). As expected for this broad-spectrum inhibitor, we found that IOX1 also inhibited TET2 in the MALDI assay – with a similar potency as 2 (IC_50_ = 2.27 ± 0.57 μM; Fig. S12†). While this inhibition and the binding mode of IOX1 in the FTO crystal structure are in line with our binding model for 2, we also suspected the latter to have attenuated chelating properties compared to IOX1, owing to the replacement of the hydroxyl group of IOX1 with the fluorine atom. This may be a favorable feature for an inhibitor, given the high conservation of this metal center in dioxygenases as a potential source of low specificity binding.

In order to gain comparable insights into the metal-coordinating abilities of both compounds, we conducted DFT-based calculations. We generated a simplified structural model by merging features of the observed coordination environments of an Fe(ii)- and α-KG-bound TET2-hmC complex (PDB-ID: 5deu) and an HIF1α complex, where a deprotonated 8HQ chelates the metal center (the latter being a square-pyramidal Zn(ii) cation; PDB-ID: 3od4). In the model, we kept the 8HQ chelate with carboxylate *trans* to the quinoline-N donor as in the HIF1α structure, but with an Fe(ii) cation in an octahedral coordination sphere complemented by a water ligand (Fig. S13†). By performing unconstrained gas-phase geometry optimizations, we computed a range of complex formation reactions with protonated and deprotonated 8HQ, 8-fluoroquinoline, quinoline and pyridine involving the replacement of one or two coordinating water molecules (Fig. S14†). While binding of the deprotonated 8HQ was found to be energetically strongly favored (−453.8 kJ mol^−1^; −4.8 kJ mol^−1^ for the unlikely protonated form), binding of 8-fluoroqinoline was energetically uphill (+14.9 kJ mol^−1^), suggesting that the latter is a significantly weaker ligand for the Fe(ii) center, however, still being somewhat stronger than a quinoline lacking the F-substituent (+43 kJ mol^−1^).

Together with our SAR and hypothesized binding model, we thus speculate that 2 may act as an isostere of IOX1 that has lower affinity to the conserved Fe(ii) center and could thus represent a novel starting point for the development of selective dioxygenase inhibitors.

## Discussion and conclusions

TET dioxygenases shape the mammalian (oxi)methylome and have been shown to be crucially involved in development and cancer disease. A number of small molecule inhibitors for controlling TET activity have been reported recently.^16–19^ However, an HTS platform for the rapid discovery, validation and characterization of small molecule inhibitors has been elusive. We present here a general approach for high-throughput screening of TET inhibitors utilizing a robust FRET assay, apply it to a 31.5k-membered compound library and identify six primary hits. In-depth validation with an orthogonal MALDI assay including resynthesis and SAR with a derivative library led to the discovery of a simple, sulfonic acid-substituted quinoline scaffold as TET2 inhibitor with low micromolar IC_50_. Docking studies suggest a plausible binding mode for this novel TET2 inhibitor, and a binding site comparison approach reveals similarities with the dioxygenase inhibitor IOX1 that is able to inhibit diverse dioxygenases *via* chelation of their conserved metal center. However, DFT calculations suggest a significantly attenuated ability of 2 to chelate Fe(ii) as a potential source of low specificity, making it an interesting candidate for the development of selective dioxygenase inhibitors as tools to study TET biology.

## Author contributions

Conceptualization, D. R. and D. S.; methodology, S. P., J. W., B. B.; software, B. B.; investigation, S. P., J. W., M. V., C. E., and G. C.; resources, R. G., T. G., S. K., and M. M.; data curation, B. B.; writing – original draft, S. P.; writing – review & editing, D. R., D. S., J. W. and G. C.; supervision, D. R. and D. S.; funding acquisition, D. R., D. S. and G. C.

## Conflicts of interest

There are no conflicts to declare.

## Supplementary Material

MD-013-D2MD00186A-s001

MD-013-D2MD00186A-s002
