# Correction: A high-throughput effector screen identifies a novel small molecule scaffold for inhibition of ten-eleven translocation dioxygenase 2

**DOI:** 10.1039/d4md90004a

**Published:** 2024-01-29

**Authors:** Shubhendu Palei, Jörn Weisner, Melina Vogt, Rajesh Gontla, Benjamin Buchmuller, Christiane Ehrt, Tobias Grabe, Silke Kleinbölting, Matthias Müller, Guido H. Clever, Daniel Rauh, Daniel Summerer

**Affiliations:** a Department of Chemistry and Chemical Biology, TU Dortmund University and, Drug Discovery Hub Dortmund (DDHD), Zentrum für Integrierte Wirkstoffforschung (ZIW) Otto-Hahn Str. 4a 44227 Dortmund Germany guido.clever@tu-dortmund.de daniel.rauh@tu-dortmund.de daniel.summerer@tu-dortmund.de

## Abstract

Correction for ‘A high-throughput effector screen identifies a novel small molecule scaffold for inhibition of ten-eleven translocation dioxygenase 2’ by Shubhendu Palei *et al.*, *RSC Med. Chem.*, 2022, **13**, 1540–1548, https://doi.org/10.1039/D2MD00186A.

The authors would like to correct the activity data reported in Table 1 and adjust the associated conclusions. Compound **2**, a simple sulfonic acid-based quinoline scaffold, was synthesized *via* hydrolysis of the corresponding sulfonic acid chloride, and an IC_50_ of 2.3 μM for the inhibition of the iron/αKG-dependent dioxygenase hTET2 was obtained from a MALDI-based inhibition assay. Compound **2** is structurally related to the known broad-spectrum iron/αKG-dependent dioxygenase inhibitor IOX-1 that features an iron-chelating 8-hydroxyquinoline (8HQ) core. In contrast, **2** bears a fluorine substituent in the 8-position that is expected to significantly attenuate iron affinity (supported by DFT calculations). This led to the conclusion that **2** acts as an isostere of IOX1 with low iron affinity, making it a starting point for TET2 inhibitors without this property. However, a later re-synthesis of **2** using a different starting material yielded an inactive compound. After consultation with the manufacturer, the authors suspect trace impurities of undefined polychlorinated quinoline species in the sulfonic acid chloride to be responsible for the observed activity, but attempts to purify these impurities proved unsuccessful. The authors thus conclude that the activity data of the compounds obtained *via* this sulfonic acid chloride are not trustable, and apologize for any confusion this data may have caused.

The authors conducted additional SAR, revealing that the sulfonic acid group of **2** can replace the carboxyl group of IOX1, leading to a simple sulfonic acid-based quinoline **18** with a virtually identical IC_50_. In contrast, a replacement of IOX1's 8-hydroxyl group with a fluorine atom led to inactivity (**19**). The authors further identified and validated other IOX1-derived compounds without the 8HQ core that nevertheless showed micromolar IC_50_ values, such as isoquinoline-3-carboxylic acid (**20**), tetrahydroisoquinoline-6,7-diol (**21**), and 3-hydroxypicolinic acid (**22**). This combined data indicates that the 8HQ core is essential for hTET2 inhibition by IOX1 itself, whereas it is not essential for IOX1-derived compounds in general, providing new starting points for TET2 inhibitors without the 8HQ core.

An updated version of Table 1 is included here.

**Table 1:** Structure–activity relationship (SAR)

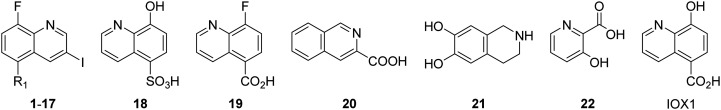					
Compound ID	R_1_	IC_50_ (μM)	Compound ID	R_1_	IC_50_ (μM)
**1**	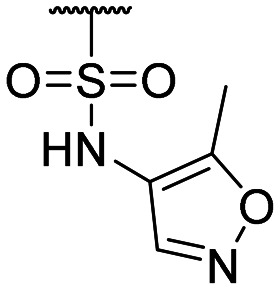	>200	**13**	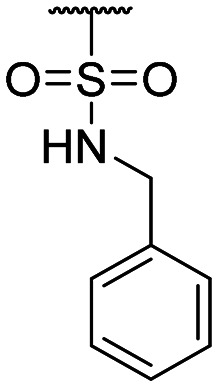	>200
**2**	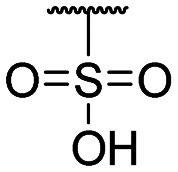	>200	**14**	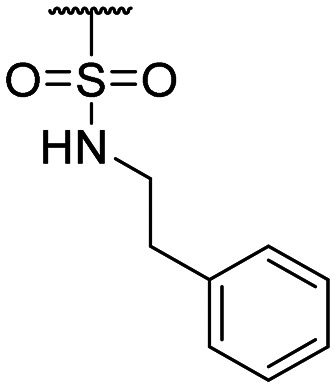	>200
**3**	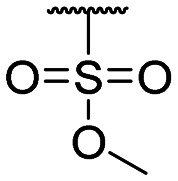	>200	**15**	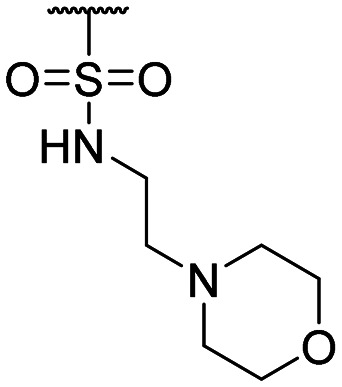	>200
**4**	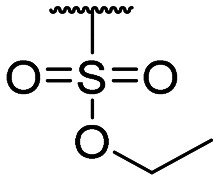	>200	**16**	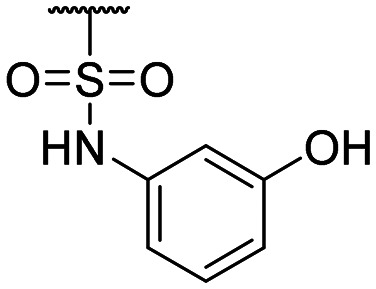	>200
**5**	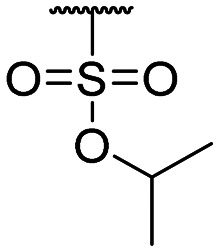	>200	**17**	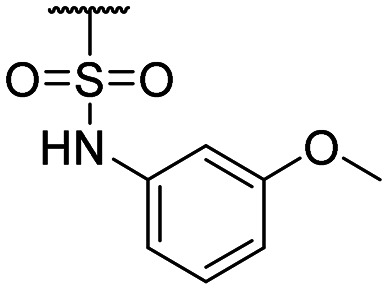	>200
**6**	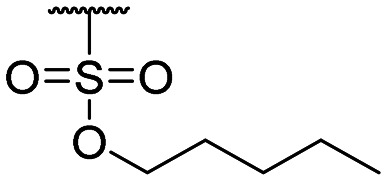	>200	**18**		12.1 ± 4.2
**7**	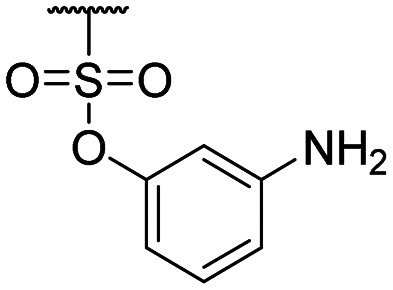	>200	**19**		>200^a^
**8**	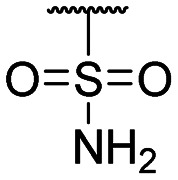	>200	**20**		170 ± 25^a^
**9**	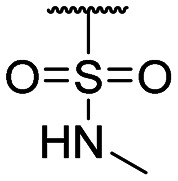	>200	**21**		8.6^a^
**10**	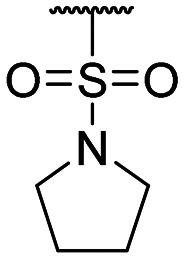	>200	**22**		48.6
**11**	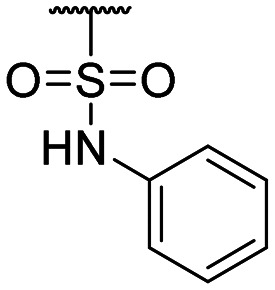	>200	IOX1		2.3 ± 0.6
**12**	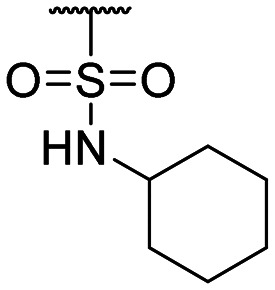	>200			

a Only one replicate was performed.

The Royal Society of Chemistry apologises for these errors and any consequent inconvenience to authors and readers.

## Supplementary Material

