# Supplementary material for: A high-throughput effector screen identifies a novel small molecule scaffold for inhibition of ten-eleven translocation dioxygenase 2
Source: RSC Med Chem. 2022 Sep 2;13(12):1540–8. doi: 10.1039/d2md00186a (PMC9749932; doi:10.1039/d2md00186a)
Supplement: MD-013-D2MD00186A-s001 [file MD-013-D2MD00186A-s001.pdf]

Electronic Supplementary Material (ESI) for RSC Medicinal Chemistry.
This journal is © The Royal Society of Chemistry 2022

Supporting Information

A high-throughput effector screen identifies a novel small molecule scaffold for inhibition of ten-eleven translocation dioxygenase 2

Shubhendu Palei[1,2], Jörn Weisner[1,2], Melina Vogt[1], Rajesh Gontla[1], Benjamin Buchmuller[1], Christiane Ehrt[1], Tobias Grabe[1], Silke Kleinbölting[1], Matthias Müller[1], Guido H. Clever[1]*, Daniel Rauh[1,]* and Daniel Summerer[1,]*

[1]Department of Chemistry and Chemical Biology, TU Dortmund University and Drug Discovery Hub Dortmund (DDHD), Zentrum für Integrierte Wirkstoffforschung (ZIW), Otto-Hahn Str. 4a, 44227 Dortmund, Germany

*Correspondence: daniel.summerer@tu-dortmund.de, daniel.rauh@tu-dortmund.de, guido.clever@tu-dortmund.de

[2]these authors contributed equally to this work

TABLE OF CONTENTS

SUPPLEMENTARY FIGURES

- Figure S1 .... S3
- Figure S2 .... S4
- Figure S3 .... S5
- Figure S4 .... S5
- Figure S5 .... S6
- Figure S6 .... S7
- Figure S7 .... S8
- Figure S8 .... S9
- Figure S9 .... S10
- Figure S10 .... S11
- Figure S11 .... S13
- Figure S12 .... S14
- Figure S13 .... S15
- Figure S14 .... S17
- Figure S15 .... S18
- Figure S16 .... S19

SUPPLEMENTARY TABLES

- Table S1: Oligonucleotides used in the study S20
- Table S2: Protein sequence of TET2 used in the study S20

MATERIAL AND METHODS S21

APPENDIX

- Plasmid Map S41

SUPPLEMENTARY REFERENCES S42

SUPPLEMENTARY FIGURES

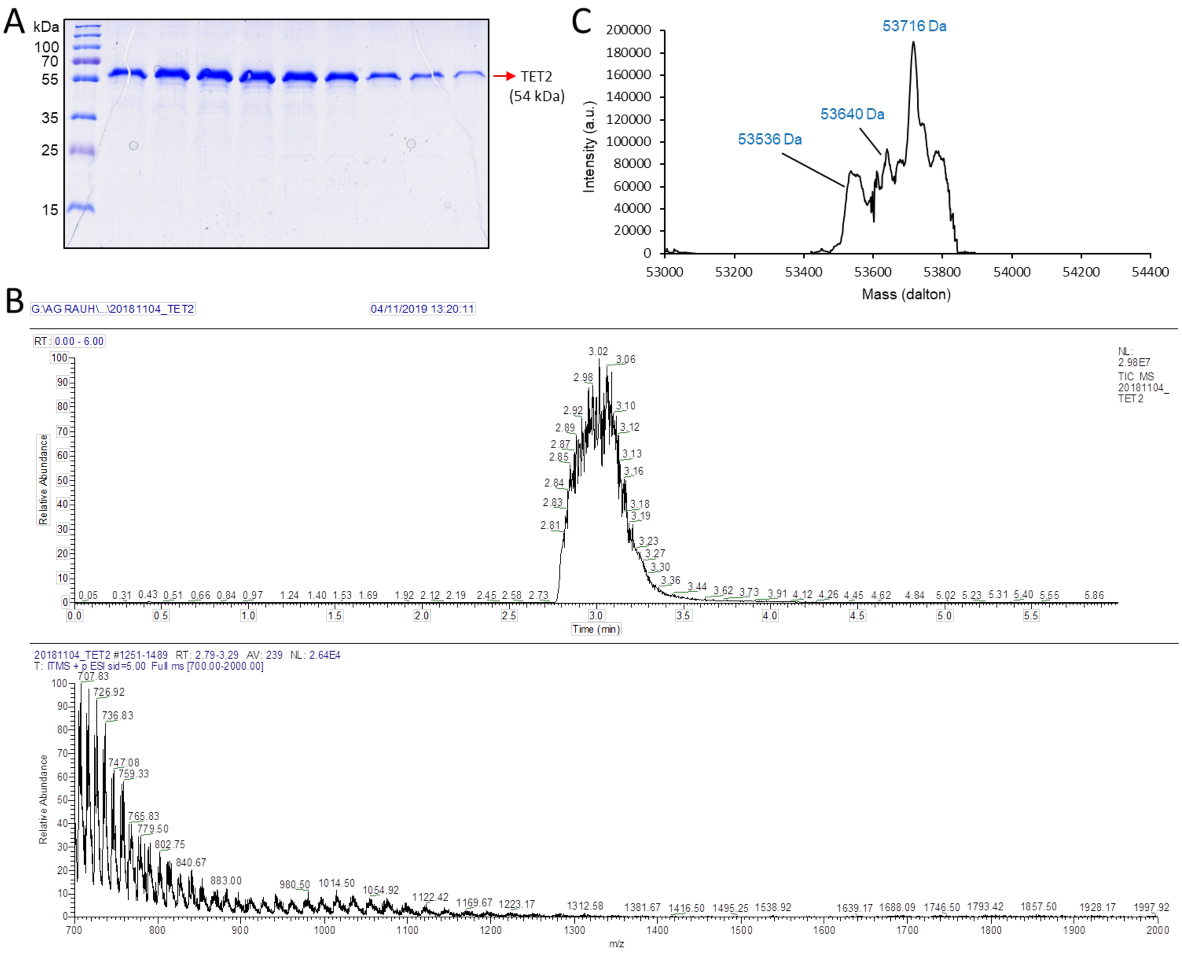

Figure S1. Characterization of purified TET2 (TET2-CD). A) SDS-PAGE analysis of the purified TET2 protein fractions from Gel Filtration with a Superdex 200 column. B) ESI-MS analysis of the purified TET2 protein. C) Deconvoluted peak of TET2 protein from ESI-MS measurement in (B). TET2: calc = 53669 Da, TET2-Met: calc = 53539 Da, found = 53536 Da, TET2-Met (gluconoylated): calc = 53717 Da, found = 53716 Da.

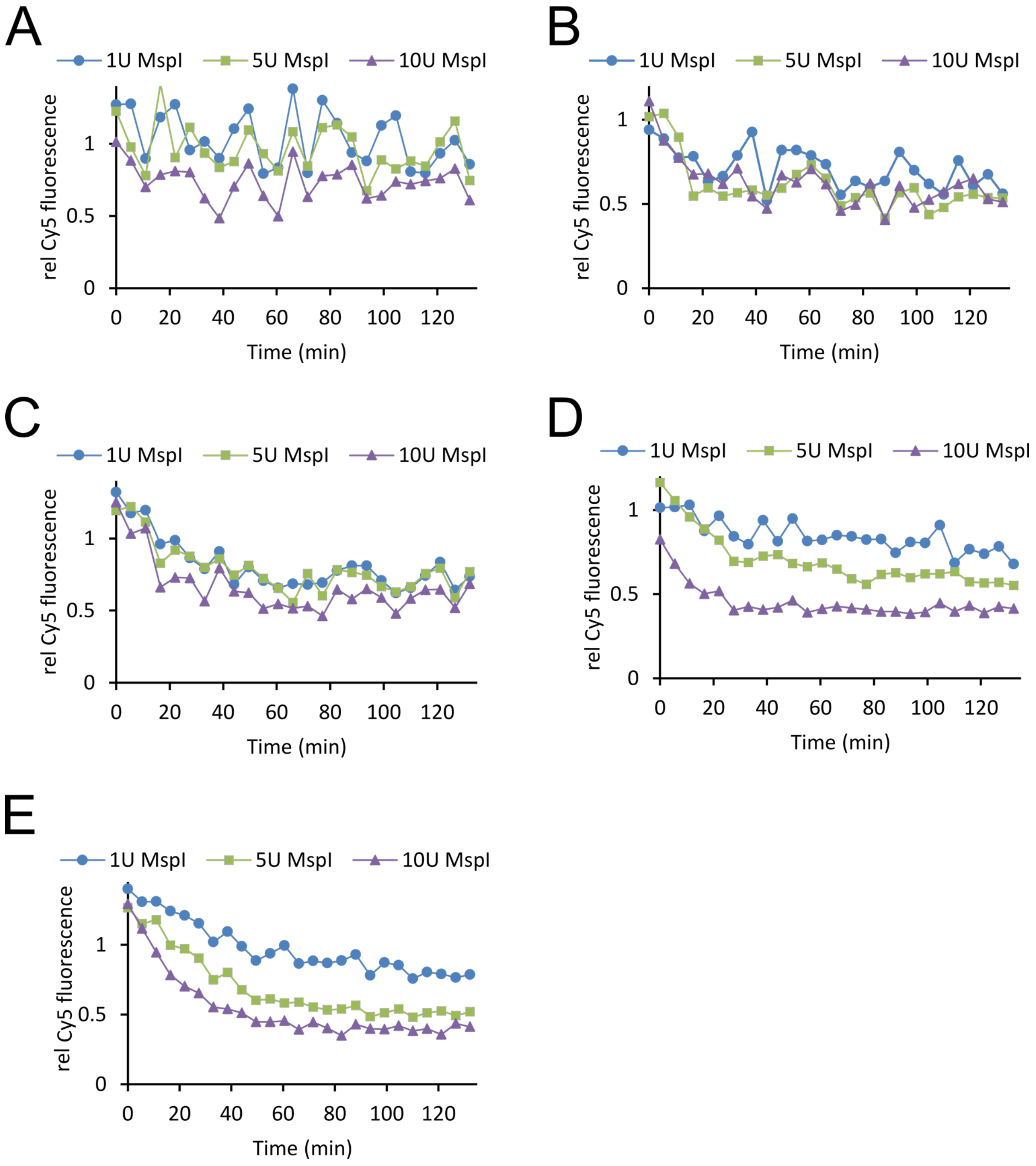

Figure S2. Optimization of duplex oligo concentration for a stable Cy5 fluorescence signal in the FRET assay. A) 0.025 µM, B) 0.05 µM, C) 0.1 µM, D) 0.25 µM, E) 0.5 µM duplex oligos treated with various concentration of *Msp*I enzyme.

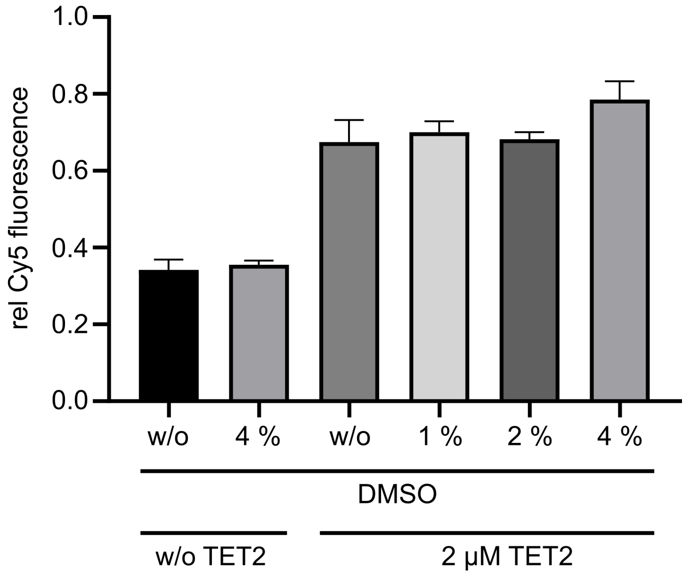

Figure S3. DMSO tolerance of TET2 and *Msp*I. Both TET2 and *Msp*I enzymes showed no compromised activity at up to 4% final DMSO concentration supporting the robustness of the FRET assay. Error bars show standard deviation of four replicate experiments.

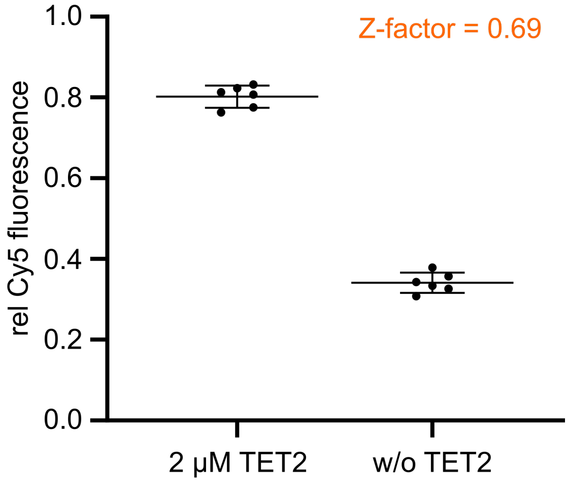

Figure S4. Z-factor (Z') for the FRET assay quantifying TET activity. Z-factor of the multiple positive (2 μM) and negative (w/o TET2) controls was calculated to be 0.69. Usual value of an excellent screening assay lies between 0.5 and 1.0.(Zhang et al., 1999). Error bars show standard deviation of six replicate experiments.

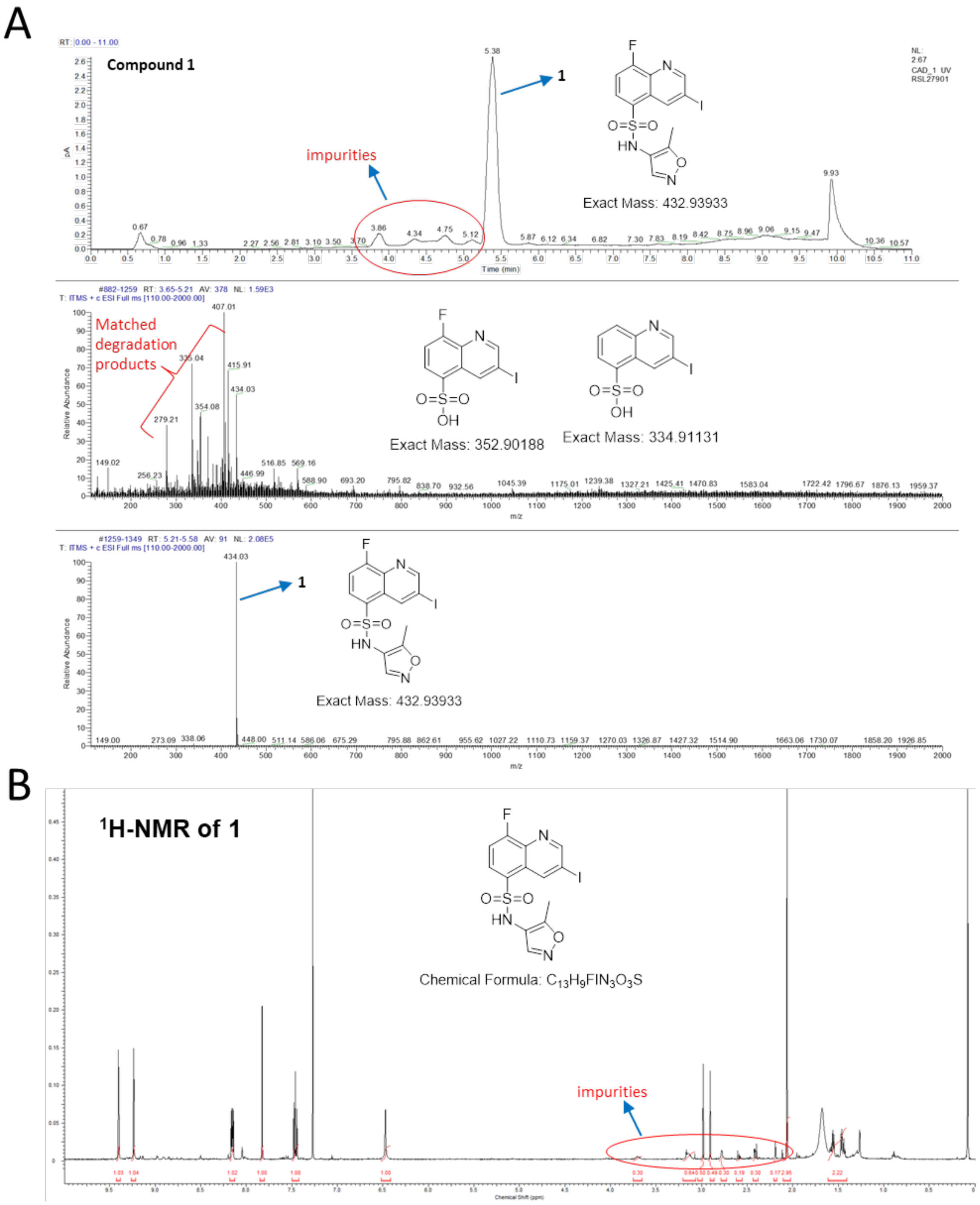

Figure S5. LC-MS analysis of commercial compound 1. A) LC-MS of the reordered compound **1** revealing minor impurities possibility resulting from the degradation of the parent compound. B) ^{1}H-NMR also confirms the presence of minor impurities in the reordered initial hit compound **1**.

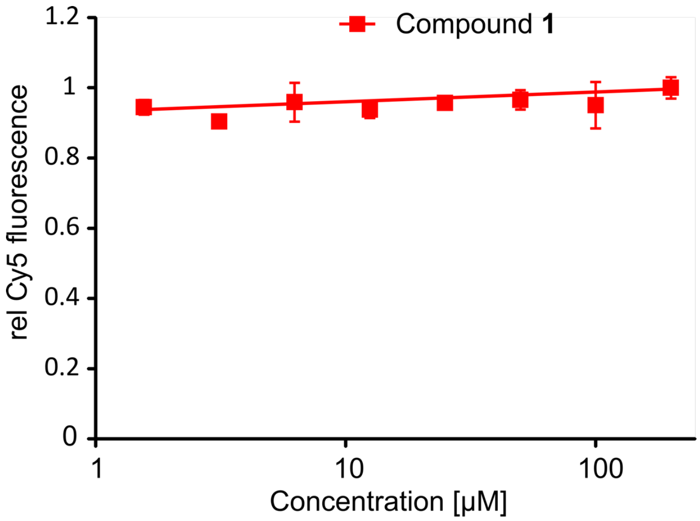

Figure S6. **Quenching of Cy5 fluorescence by compound 1.** No change in Cy5 fluorescence was observed with a range of inhibitor concentration in the absence of *Msp*I. Error bars show standard deviation of two replicate experiments.

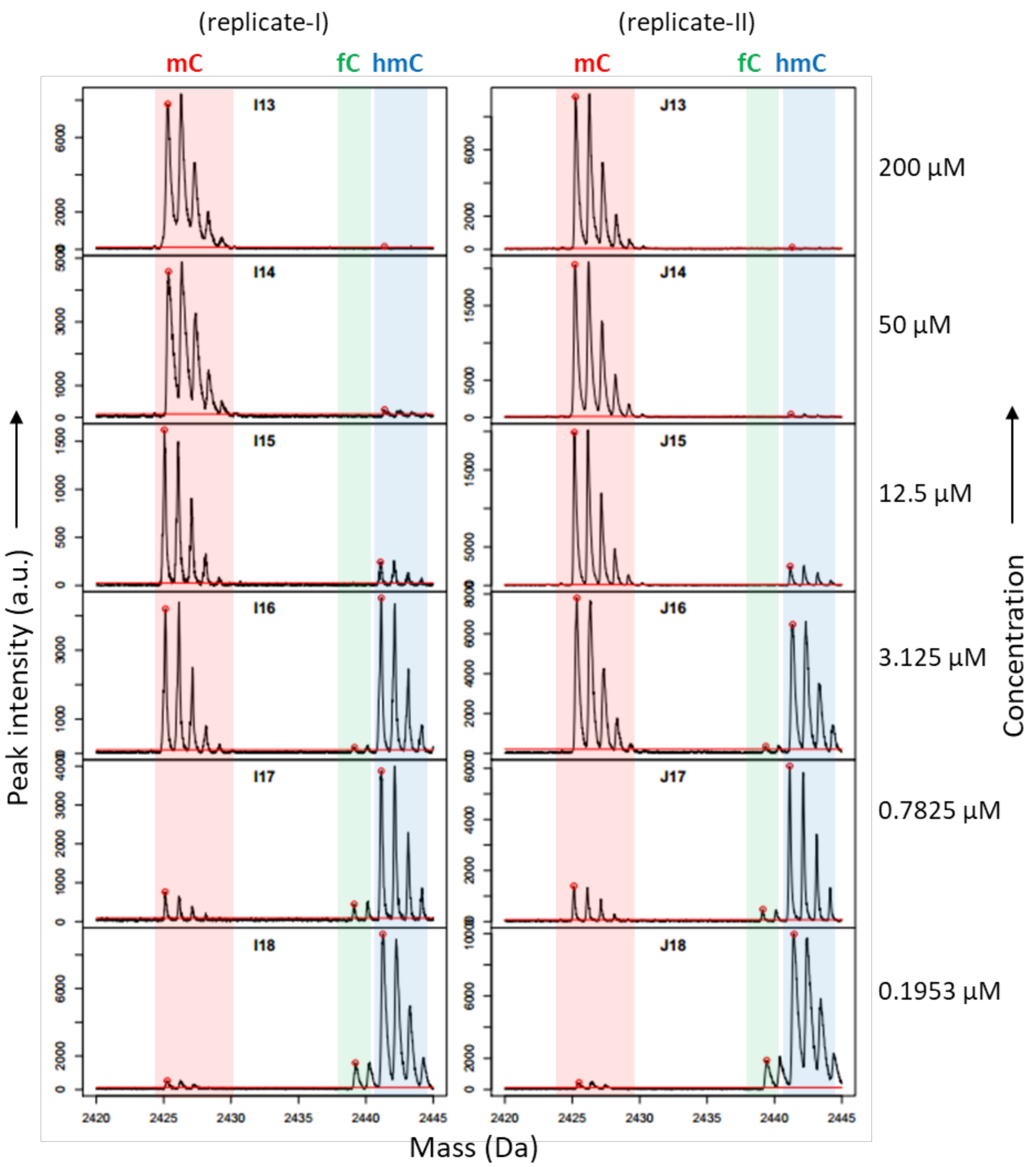

Figure S7. Representative MALDI peaks of the dose response of the initial hit compound 1. $[M+H]^+$ were calculated for mC, fC and hmC to be 2425 Da, 2439 Da and 2441 Da respectively.

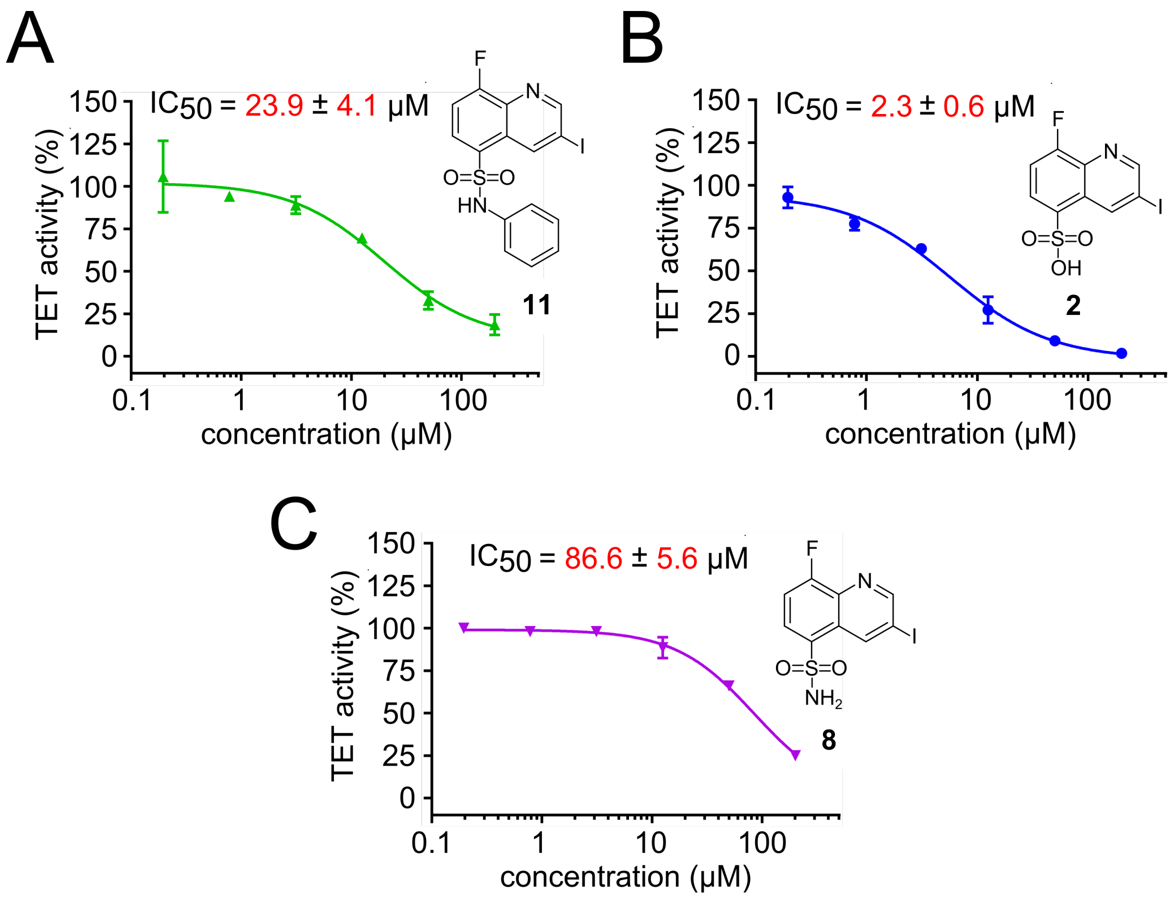

Figure S8. Dose response curves of **11** (A), **2** (B) and **8** (C) as determined with the MALDI assay. Error bars showing the standard deviation from two technical replicate experiments. IC50 values are obtained from three independent experiments with technical duplicates each.

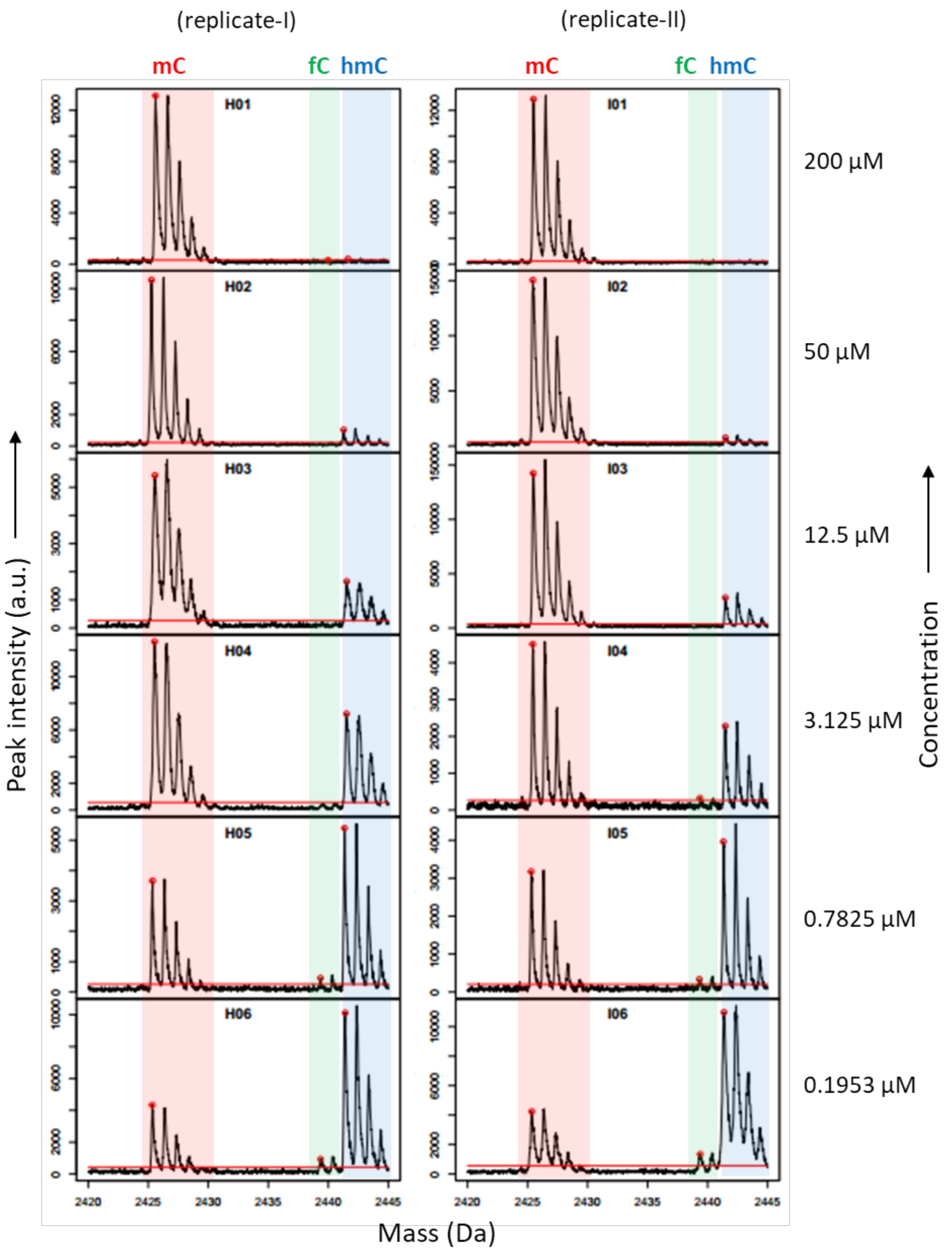

Figure S9. Representative MALDI peaks of the dose response of compound 2. $[M+H]^+$ were calculated for mC, fC and hmC to be 2425 Da, 2439 Da and 2441 Da respectively.

A

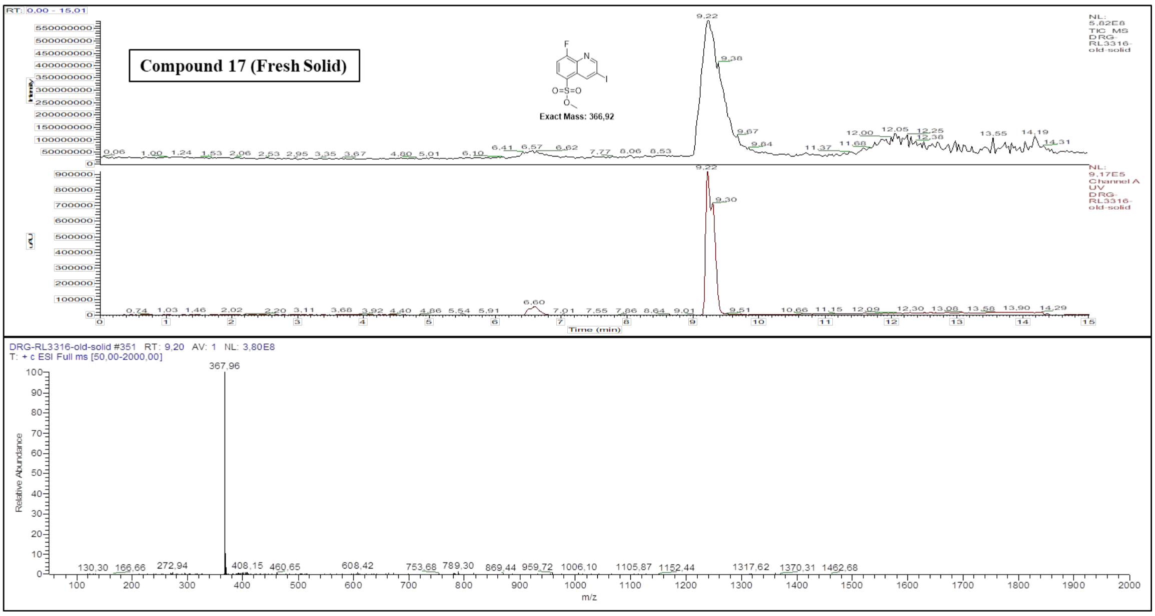
Compound 17 (Fresh Solid)
Exact Mass: 366,92
DRG-RL3316-old-solid #351 RT: 9,20 AV: 1 NL: 3,80E8
T: + c ESI Full ms [50,00-2000,00]
Time (min)
m/z
Relative Abundance

B

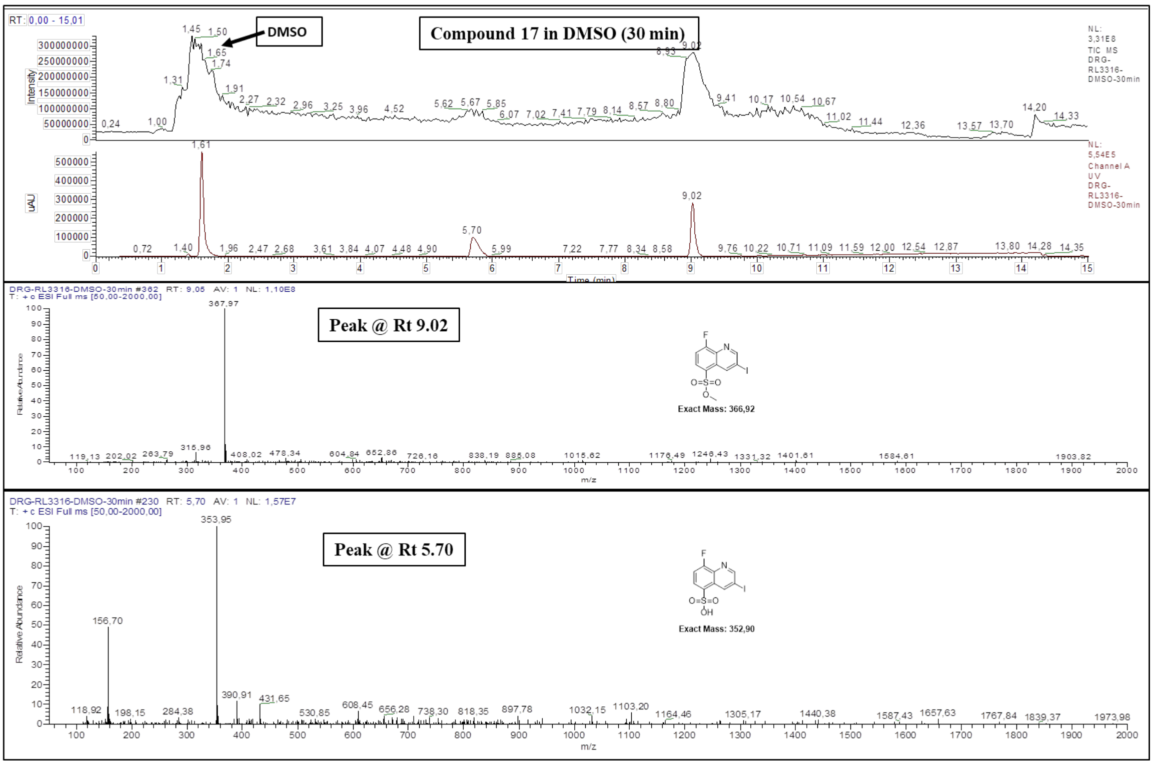
DMSO
Compound 17 in DMSO (30 min)
DRG-RL3316-DMSO-30min #362 RT: 9,05 AV: 1 NL: 1,10E8
T: + c ESI Full ms [50,00-2000,00]
Peak @ Rt 9.02
Exact Mass: 366,92
DRG-RL3316-DMSO-30min #230 RT: 5,70 AV: 1 NL: 1,57E7
T: + c ESI Full ms [50,00-2000,00]
Peak @ Rt 5.70
Exact Mass: 352,90
m/z
Relative Abundance

C

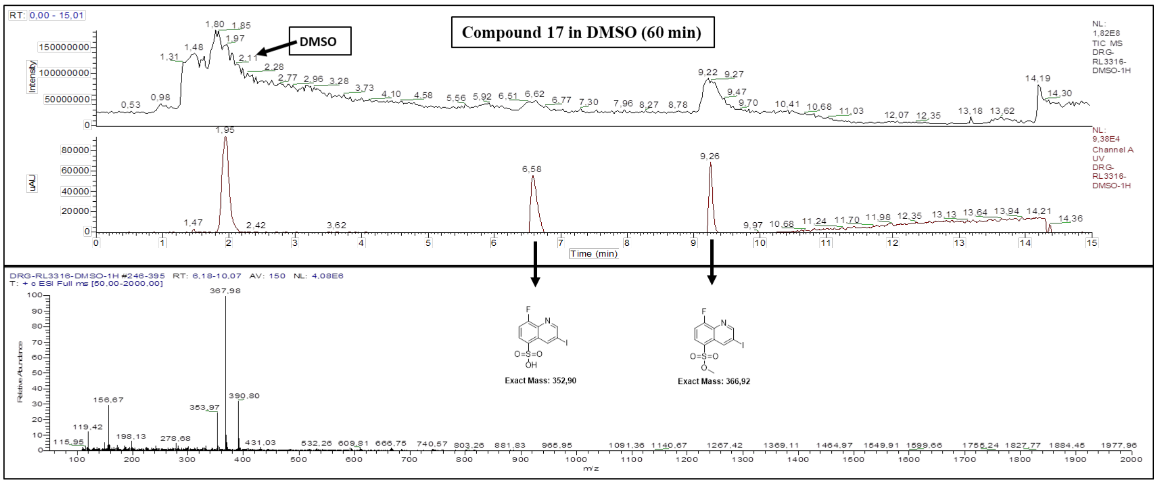

D

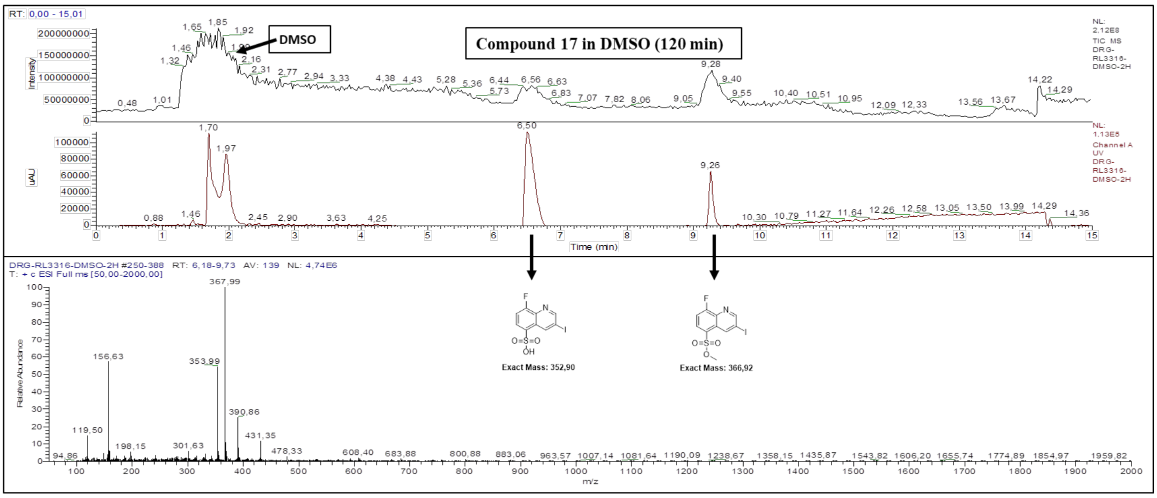

E

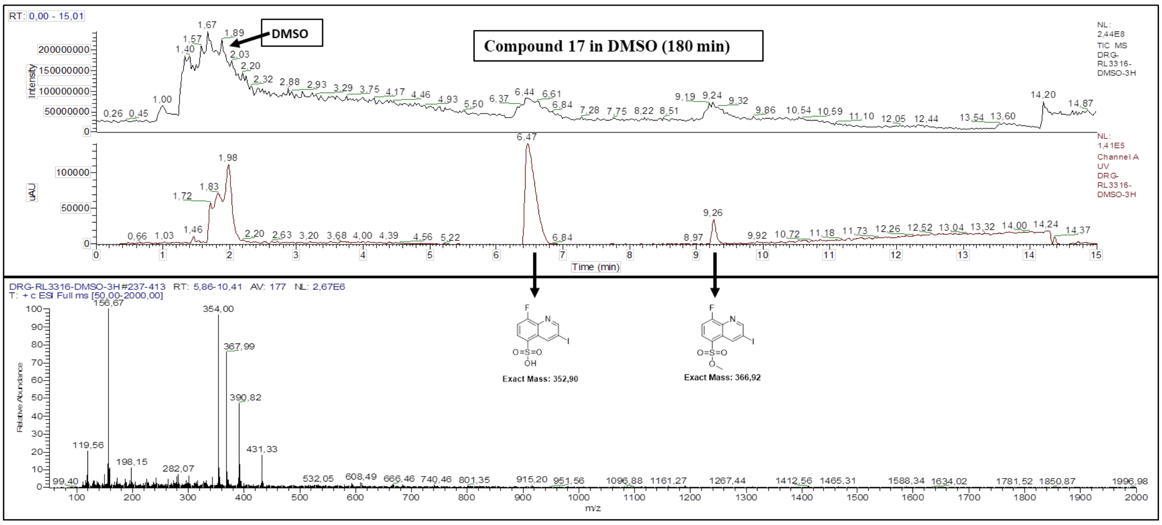

Figure S10. **Hydrolytic stability of methyl sulfonic ester 3**. LC-MS characterization of methanol/DMSO stock in PBS incubated at 37°C for A) 0 min, B) 30 min, C) 1 hour, D) 2 hours, E) 3 hours.

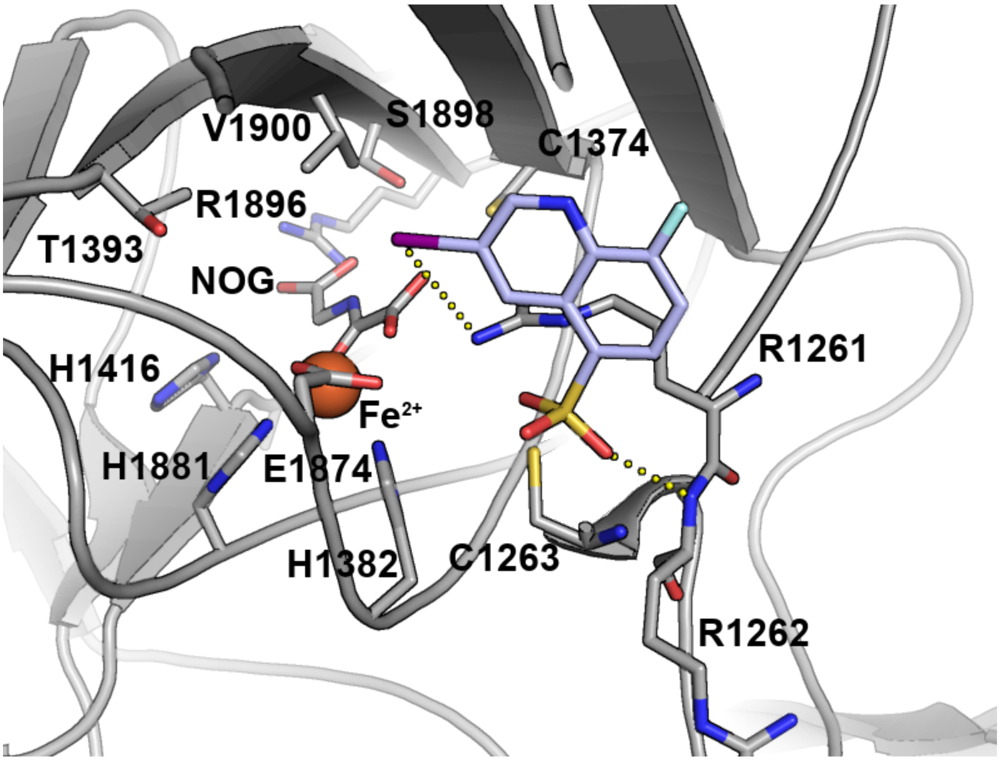

Figure S11. Predicted highest-scored binding mode of 2 after a molecular docking in the 5mC binding site. The binding site visualizations were generated using UCSF Chimera(Pettersen et al., 2004).

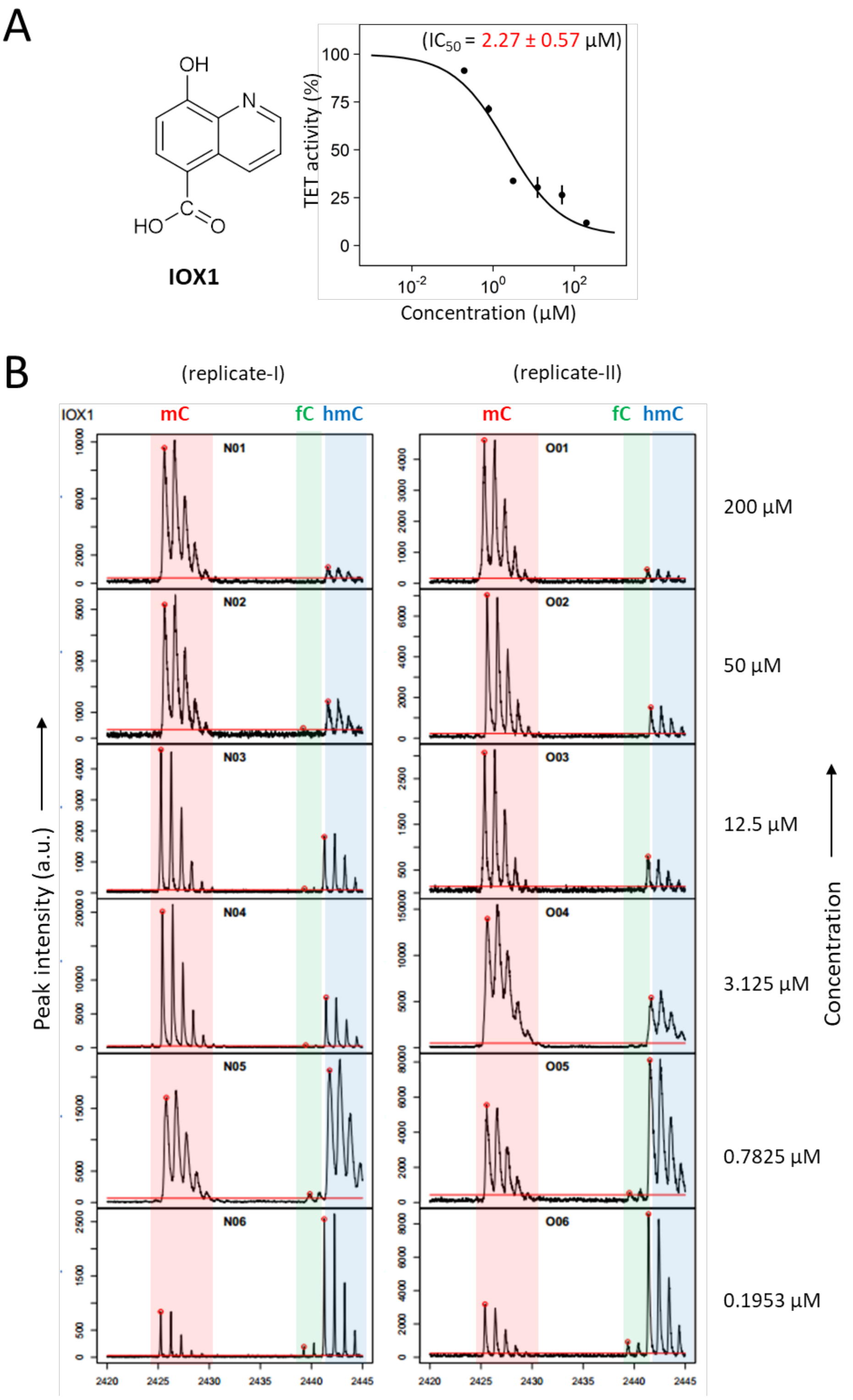

Figure S12. **Activity of IOX1 inhibitor.** A) Dose response and IC_{50} of IOX1 inhibitor of TET2. Error bars showing the standard deviation from two technical replicate experiments. IC50 values are obtained from three independent experiments with technical duplicates each. B) Representative MALDI peaks of dose response of IOX1. $[M+H]^+$ were calculated for mC, fC and hmC to be 2425 Da, 2439 Da and 2441 Da respectively.

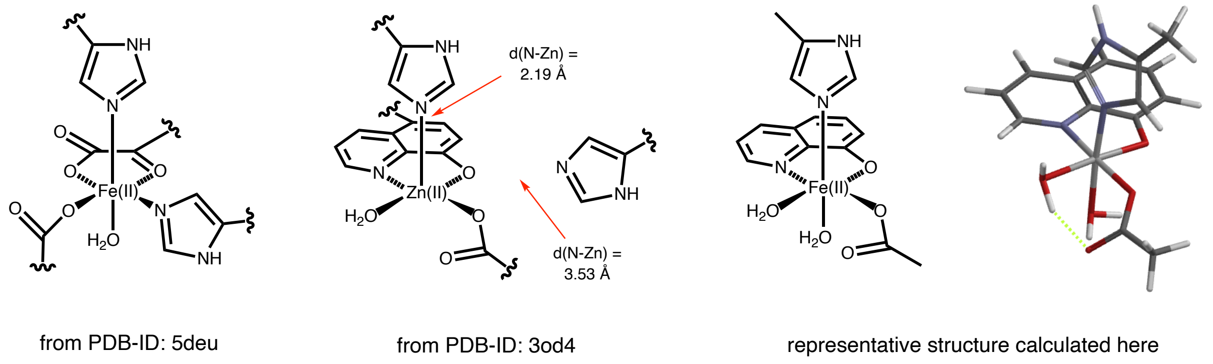

Figure S13. **Structural model for DFT calculations.** Left: two structures showing the coordination environments of metal centers found in the X-ray structures of Fe(II)- and 2-oxoglutarate-binding TET2-hmC complex (PDB-ID: 5deu) and of the hypoxia-inducible factor 1-alpha inhibitor (PDB-ID: 3od4) where a deprotonated 8-hydroxyquinoline ligand chelates the metal center, the latter being a square-pyramidal Zn(II) cation. Right: structural model for DFT calculations generated by merging features of both observed coordination environments by keeping the 8-hydroxyquinoline chelate with carboxylate *trans* to the quinoline-N donor as in PDB-ID: 3od4, but containing an iron(II) cation whose octahedral coordination sphere is complemented by a water ligand *trans* to the axial imidazole as in PDP-ID: 5deu. For computational details see section 12.

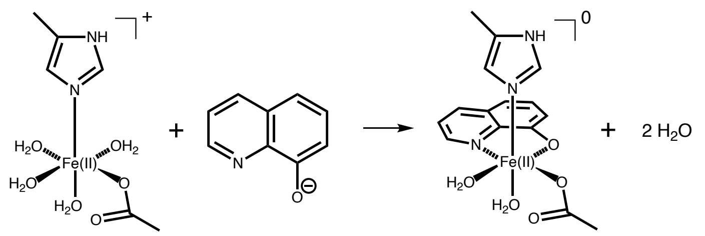

ΔE = –453.8 kJ/mol

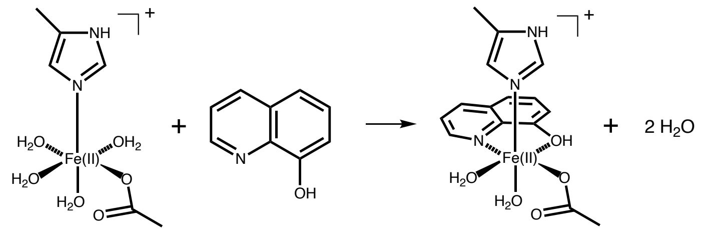

ΔE = –4.8 kJ/mol

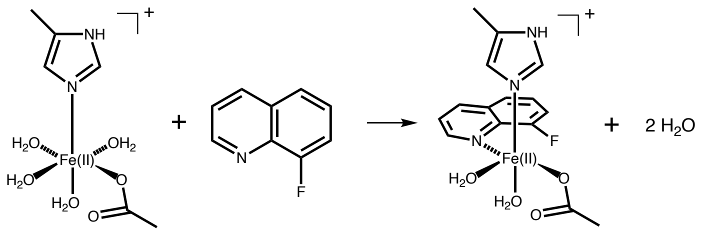

ΔE = 14.9 kJ/mol

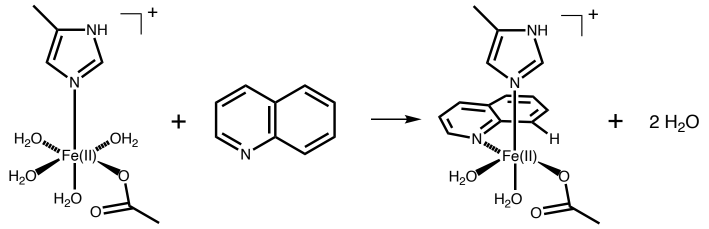

ΔE = 43.0 kJ/mol

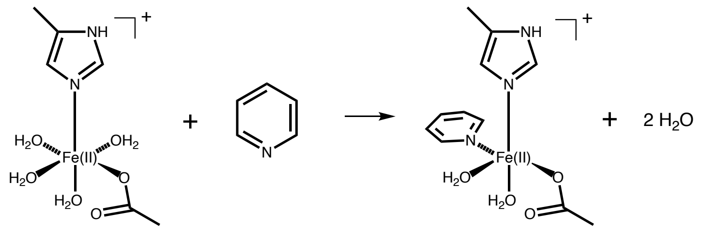

ΔE = 52.2 kJ/mol

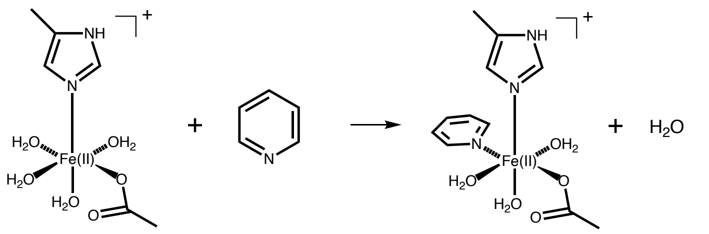

ΔE = –56.5 kJ/mol

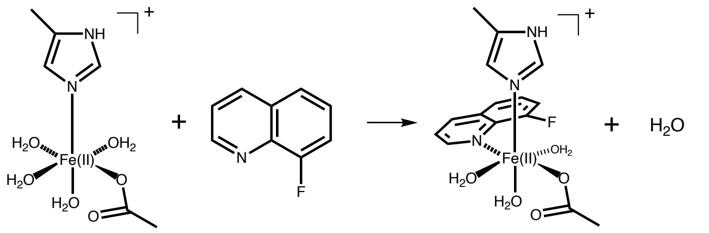

ΔE = –45.6 kJ/mol

Figure S14. **Computed complex formation reactions**. Coordination of deprotonated 8HQ to the cationic starting complex, here chosen to carry water ligands in all positions not occupied by amino acid side chain-derived ligands, is energetically strongly favored (–453.8 kJ/mol) as was expected for the potent chelate ligand carrying a negative charge. Coordination of non-deprotonated 8-hydroxyquinoline would only lead to a small energy release (–4.8 kJ/mol). In comparison, when coordinating the 8-fluoroquinoline motif of **2** in the same position, likewise replacing two water ligands, the process proceeds energetically uphill by 14.9 kJ/mol. While this already indicates that 8-fluoroquinoline is a significantly less attractive ligand for the Fe(II) center, the following computations show, that the fluorine substituent could still contribute a small stabilizing effect to the complex. Coordination of quinoline (with a hydrogen substituent instead of the fluorine) is energetically even less favored (+43.0 kJ/mol) and so is the coordination of pyridine in the hypothetical situation that it replaces two adjacent water molecules (+52.2 kJ/mol). Coordination of the rather small pyridine ligand under replacement of only one water molecule, however, is again going along with energy release (–56.5 kJ/mol), as would be expected from a coordination chemistry point of view. Coordination of 8-fluoroquinoline under replacement of only one water molecule seems energetically feasible, but results in an unlikely complex geometry (see Fig. S17).

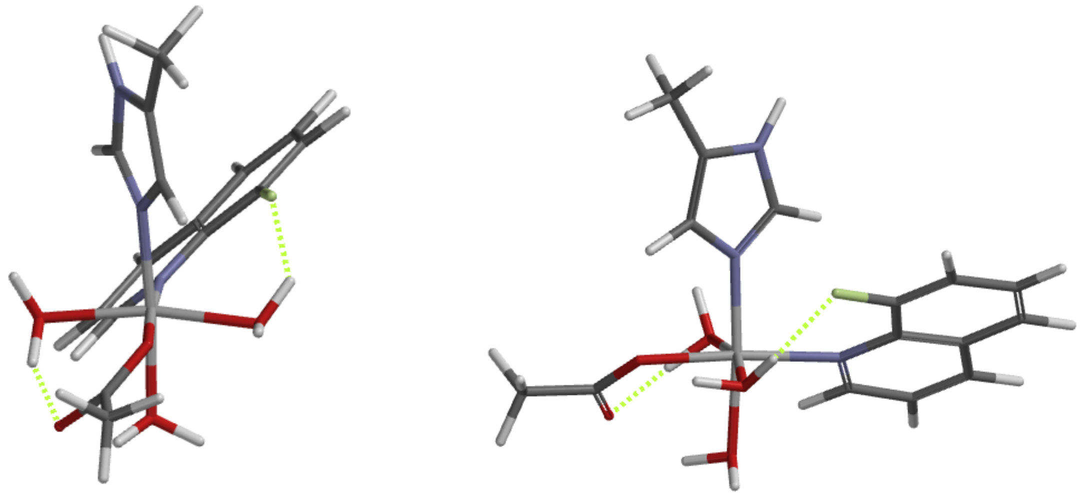

Figure S15. **Alternative binding mode of 8-fluoroquinoline with only one water molecule replaced by the coordinating nitrogen donor (see Fig. S16, last line)**. In this structure, the quinoline unit twists so that the fluoro substituent adopts a position in the octahedron's octant spanned by $N_{quinoline}$, $N_{imidazole}$ and a water molecule (and hydrogen bonds to the latter). While formation of this complex is computed to be energetically feasible (–45.6 kJ/mol) in the gas phase, steric pressure of the squeezed-in 8-fluoroquinoline ligand leads to a significant deviation of the Fe(II) complex geometry from the expected octahedral shape with $N_{quinoline}$-Fe-$O_{acetate}$ angle = 175.6°, $N_{imidazole}$-Fe-OH_2 angle = 174.5° and in particular a H_2O-Fe-OH_2 angle = 166.4°.

It is further worth noting, that a search in the Chemical Database SciFinder did not yield any iron complexes containing an N-coordinated 8-fluoroquinoline and a search in the Cambridge Crystal Structure Database (CCSD) for substructures of octahedral transition metal complexes containing an N-coordinated 8-fluoroquinoline did also not reveal any hit.

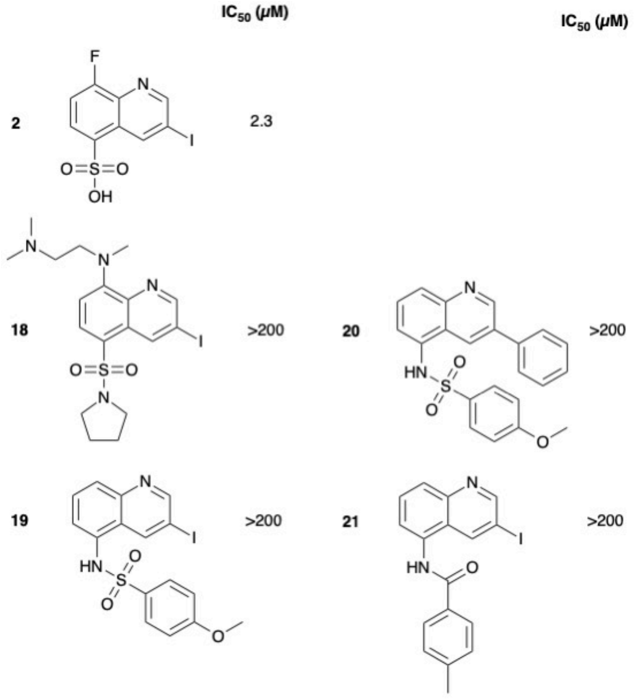

Figure S16. **IC50 values of MALDI assay for compound 2 and additional compounds 18-21 with variations at the fluorine and iodine positions.**

Supplementary Tables

Table S1: Oligonucleotides used in this study

Name	Sequence (5' → 3')
o3260	Cy3-GGGAC(mC)GGAGGG-Cy5
o3261	CCCTCCGGTCCC
o4091	CAC(mC)GGTG

Table S2: Protein sequence of TET2 used in this study

Protein (plasmid)	Sequence (6xHis-thrombin site-hTET2 CD (LC insert deleted))
**hTET2CD (pET15b)**	MGSSHHHHHHSSGLVPRGSHMGGSDFPSCRCVEQIIEKDEGPFYTH LGAGPNVAAIREIMEERFGQKGKAIRIERVIYTGKEGKSSQGCPIAK WVVRRSSSEEKLLCLVRERAGHTCEAAVIVILILVWEGIPLSLADKL YSELTETLRKYGTLTNRRCALNEERTCACQGLDPETCGASFSFGCS WSMYYNGCKFARSKIPRKFKLLGDDPKEEEKLESHLQNLSTLMAPT YKKLAPDAYNNQIEYEHRAPECRLGLKEGRPFSGVTACLDFCAHA HRDLHNMQNGSTLVCTLTREDNREFGGKPEDEQLHVLPLYKVSDV DEFGSVEAQEEKKRSGAIQVLSSFRRKVRMLAEPVKTCRQRKLEAK KAAAEKLSGGGGSGGGGSGGGGSDEVWSDSEQSFLDPDIGGVAVA PTHGSILIECAKRELHATTPLKNPNRNHPTRISLVFYQHKSMNEPKH GLALWEAKMAEKAREKEEECEKYG

MATERIALS & METHODS

1. Plasmid and cloning:

A TET2 construct was designed as previously described by Hu et al (Hu et al., 2013) spanning the C-terminal catalytic domain (1129-1936aa). To facilitate expression in *E. coli*, the low-complexity insert (1481-1843aa) which is predicted to be unstructured in solution was replaced by a GS-linker. The corresponding gene was codon-optimized for *E. coli* expression, synthesized and subsequently subcloned into a pET-15b vector succeeding a His_6-*tag* and thrombin cleavage site by using the *Nde*I and *Xho*I restriction sites (Geneart AG, Germany).

2. Protein expression and purification:

The plasmid was transformed into chemically competent BL21 (DE3) *E. coli* cells by heat shock and subsequently selected on LB agar containing 100 µg/mL ampicillin by incubation at 37 °C overnight. The next day, the transformed clones were washed off with 5 mL TB medium, transferred to additional 100 mL TB medium containing 100 µg/mL ampicillin and grown at 37 °C and 150 rpm overnight. Subsequently, 10 L of TB medium containing 100 µg/mL ampicillin were inoculated 1:100 with the starter culture and grown to OD_{600} = 0.8 at 37 °C, 150 rpm before target protein expression was induced by addition of 0.5 mM IPTG. The expression culture was shaken for additional 16 h at 18 °C, 150 rpm followed by centrifugation at 4000 x g, 4 °C for 20 min. The cell pellet was snap frozen in liquid nitrogen and stored at -80 °C. The cell pellet was resuspended in buffer A (50 mM Tris, 500 mM NaCl, 1 mM DTT, 5% glycerol, pH 8.0) and lysed using a microfluidizer. The lysate was cleared by centrifugation at 4000 x g, 4 °C for 60 min and the resulting supernatant was loaded onto a hand-packed 20 mL Ni-NTA column (Ni-NTA Superflow, Qiagen) pre-equilibrated with buffer A. Afterwards, the column was washed with 10 column volumes (CV) buffer A and nonspecifically bound proteins were eluted with 4% buffer B (50 mM Tris, 500 mM NaCl, 500 mM imidazole, 1 mM DTT, 5% glycerol, pH 8.0). Finally, the target protein was eluted using a gradient from 4% up to 50% of buffer B over 30 min. Fractions containing the His_6-tagged TET2 were pooled, diluted 1:20 with buffer C (50 mM Tris, 10 mM NaCl, 1 mM DTT, 5% glycerol, pH 8.0) and loaded onto an anion-exchange chromatography column (5 mL HiTrap Q FF, GE Healthcare) pre-equilibrated with buffer C. The resin was washed with 10 CV buffer C and a gradient up to 30% of buffer D (50 mM Tris, 1000 mM NaCl, 1 mM DTT, 5% glycerol, pH 8.0) was used to elute bound proteins. Fractions containing the target protein were pooled, concentrated to approximately 5 mg/mL

using centrifugal concentrators (Vivaspin 20 MWCO 30 kDa, Sartorius) and further purified using a size-exclusion chromatography column (HiLoad 16/600 200 pg, GE Healthcare) equilibrated with buffer E (20 mM HEPES, 100 mM NaCl, 1 mM DTT, 5% glycerol, pH 7.5). The protein was concentrated to approximately 5 mg/mL, snap-frozen in liquid nitrogen and stored at -80 °C. The identity and the purity of the protein was determined by electrospray ionization mass spectrometry (Velos Pro Dual-Pressure Linear Ion Trap Mass Spectrometer, Thermo Fisher Scientific).

3. LC-MS analysis of purified protein:

The His_6-tagged TET2 protein at a concentration of 1.0 mg/mL was analyzed by ESI-MS using a Thermo Fisher Scientific Dionex UltiMate 3000 HPLC system connected to a Thermo Fisher Scientific Velos Pro (2d ion trap). 1 µL of the sample was injected and separated using a AdvanceBio Desalting-RP cartridge starting at 5% of solvent B for 5 min, followed by a gradient up to 80% of solvent B over 2.5 min with a flow rate of 400 µL/min with 0.1% formic acid in water as solvent A and 0.1% formic acid in MeCN as solvent B. A mass range of 700–2000 m/z was scanned, and raw data were deconvoluted and analyzed with MagTran software(Zhang and Marshall, 1998).

4. FRET assay for quantification of i*n vitro* TET activity:

The FRET assay was performed in microplates (384 and 1536 well, flat bottom, black, polystyrene, small volume, Greiner). The pipetting was done with hand (for assay validation) or the help of the Multidrop™ Combi Reagent Dispenser (for screening the compound library) and the Echo 520 Liquid Handler (for compound dilution and transfer). The fluorescence signals were recorded on an Infinite® M1000 (Tecan).

For *in vitro* enzymatic activity assays, 0.5 µM of double-stranded DNA substrates (annealed oligos o3260 and o3261) were incubated with a fixed (2 µM) or variable concentrations of TET2 in assay buffer (50 mM potassium acetate 20 mM Tris-acetate, 10 mM magnesium acetate, 100 µg/mL BSA, 2 mM sodium ascorbate, 1 mM DTT, 1 mM α-KG, pH 7.0) and in presence of inhibitor or DMSO as a control. The mixture was incubated in a humidified chamber for 30 minutes at RT followed by addition of 75 µM ammonium Fe(II)-sulfate at 37 °C for 1 hour. Afterwards, *Msp*I (1-20 U) was added to the reaction mixture and incubated for the appropriate time at 25 °C in a humidified dark chamber. The excitation wavelength for Cy3 was set to 552 nm and the emission wavelength for Cy5 to 665 nm.

For biochemical characterization, the hits from the screen and the compounds from the virtual screen were added to the DNA-TET2-solution as serial dilutions (8 concentrations, 2-fold dilution, 200 µM highest concentration) with the Echo 520 Liquid Handler. The reaction mixtures were preincubated for 1 hour before the iron solution was added.

5. High-Throughput inhibitor screening of small molecule library:

The screening of 31500 compounds was performed using the in-house RASPELD unit (Robotics-Assisted Screening Platform for Efficient Ligand Discovery)(Wolle et al., 2018). First, 5 µL of assay solution (50 mM potassium acetate 20 mM Tris-acetate, 10 mM magnesium acetate, 2 µM TET2, 0.5 µM 5mC-dsDNA, 1 mM 2-oxoglutarate, 1 mM DTT, 2 mM sodium ascorbate, 100 µg/mL BSA, pH 7.0) were transferred into each well of a black 1536-well microplate (Greiner Bio-One) using a Multidrop™ Combi Reagent Dispenser (Thermo Fisher Scientific) followed by centrifugation of each plate at 200 x g for 1 min. Afterwards, 27.5 nL per compound were added into the respective destination wells using the Echo 520 liquid handler (Labcyte) to reach a final concentration of 55 µM. DMSO and DFOA were added to 16 wells per plate and used as negative and positive controls, respectively. The plates were shaken at 1000 rpm for 30 s, centrifuged at 200 x g for 1 min, and incubated in a humidified dark chamber at room temperature for 1 h. The TET2-mediated oxidation of 5mC-dsDNA was initiated by addition of 0.5 µL 825 µM Fe(II)-sulfate (ammonium iron(II) sulfate in ddH_2O) to a final concentration of 75 µM followed by shaking at 1000 rpm for 30 s, centrifugation at 200 x g for 1 min, and incubation in a humidified dark chamber at 37 °C for 1 h. Afterwards, 5 µL of *Msp*I (1 U in 50 mM potassium acetate 20 mM Tris-acetate, 10 mM magnesium acetate, 100 µg/mL BSA, pH 7.0) were added to each well and the Cy5 fluorescence was recorded on an Infinite M1000 plate reader (Tecan) immediately after (t_0). After an additional incubation for 4 h at room temperature, the Cy5 fluorescence was recorded again (t_{240}). Relative Cy5 fluorescence was calculated as the ratio of Cy5 fluorescence intensities at t_{240} and t_0. The Z-factor, a statistical parameter reflecting assay robustness and its suitability for high-throughput screening (Zhang *et al.*, 1999) was determined as follows:

$$Z' = 1 - \left(3 \cdot \frac{\sigma_{pos} + \sigma_{neg}}{|\mu_{pos} - \mu_{neg}|}\right)$$

σ: standard deviation, μ: mean, pos: positive control, neg: negative control

6. Hit validation and IC_{50} determination using orthogonal semi-high throughput MALDI assay:

The MALDI assay was performed in microplates (384 well, flat bottom, white, polystyrene, small volume, Greiner). The pipetting was done with hand (for assay validation) or the help of the Multidrop™ Combi Reagent Dispenser (for determining dose response of the SAR compounds) and the Echo 520 Liquid Handler (for compound dilution and transfer).

For *in vitro* enzymatic activity assays, 5 µL of 2 µM of TET2 in 2x assay buffer (50 mM HEPES, 100 mM NaCl, 2 mM Sodium Ascorbate, 1 mM α-KG, 1 mM DTT, pH 7.4) was incubated with a serially diluted concentration (0.1953-200 µM) of inhibitor or DMSO as a control in a humidified chamber for 30 minutes at RT followed by addition of 5 µL of 1 µM annealed palindromic duplex oligo (o4091) in water and 75 µM of ammonium Fe(II)-sulfate in water (2x dilution). The plate was sealed to stop evaporation and incubated at 37 °C for 1 hour for TET oxidation (final concentrations of oligo and TET2 are 0.5 µM and 1 µM respectively). Afterwards, the plate was cooled at RT for 5-10 minutes and centrifuged briefly.

For desalting of the sample for MALDI analysis, 10 µL aqueous slurry (50%) cation exchange resins (BioRad) was added to the reaction mixture. Beforehand, the cation exchange resins were washed with deionized water (3 times) followed by equilibration with 1M ammonium bicarbonate solution. Finally, the equilibrated resins were washed with deionized water (3 times) before being added to the reaction mixture for desalting.

The desalted assay plate was briefly centrifuged and 1 µL of the supernatant was spotted onto a solidified matrix of 50 mg/mL 3-hydroxypicolinic acid in 50% acetonitrile aq / 0.1% TFA aq and 10 mg/mL diammonium hydrogen citrate on a ground steel MTP 384 target. Spectra were recorded in linear positive mode on a Bruker ultrafleXtreme MALDI.TOF/TOF system.

7. Processing of MALDI mass spectra and IC_{50} curve fitting:

The recorded MALDI spectra were processed with a custom script (part of the summerrmass R package available under https://zenodo.org/record/5501758; pipeline template-A01.R) to extract the desired peak heights and determine the IC_{50} for each compound with given additional metadata and internal controls. In brief, Bruker FID files were converted to mass spectrometry XML (mzXML) using CompassXport v3.0.4 (Bruker Daltonik GmbH, Bremen, Germany) and processed in R v4.0.5(R: A language and environment for statistical

computing., 2021) using functions implemented in the packages MALDIquant (Gibb and Strimmer, 2012) and MALDIquantForeign(Gibb, 2019). Specifically, the spectra baselines were removed using the statistics-sensitive non-linear iterative peak-clipping (SNIP) algorithm before spectra of replicate measurements of the same well (if present) were aligned using the default locally weighted scatterplot smoothing (lowess) implemented in MALDIquant. Replicate spectra were then averaged taking the arithmetic mean and trimmed to m/z between 2420 and 2445. Peak intensities were extracted at 2425 (mC), 2441 (hmC), and 2439 (fC) m/z at a tolerance of 0.5 m/z units. To identify the peaks, spectra were smoothed using the Savitzky-Golay algorithm at a half-window size of 0.5 divided by the spectral resolution and the m/z peak closest to the smoothed peak in the unsmoothed spectrum was taken. For each sample, the proportion of mC, hmC and fC intensity of the total peak intensity of these three ions was calculated assuming that under the same experimental treatment, the ionization of each ion was similar(Sappa et al., 2021). The extracted fractional peak intensity was merged with data on compound identity and compound concentration in an automated manner and the decay of educt concentration (100 – % of mC) was fitted against compound concentration using a four-parameter log-logistic dose-response model implemented in the drc package.(Ritz et al., 2016) Typically, two independent technical replicates of the compound dilution series were assessed per fitted model. Model estimates and fitted curves were visualized with broom and ggplot2 packages of R's tidyverse(Wickham, 2019).

8. Re-synthesis of the SAR analogues:

General details:

All reagents and solvents were purchased from Enamine, Acros, Activate Scientific, Alfa Aesar, Apollo Scientific, Merck, Sigma-Aldrich, TCI Chemicals or VWR and used without further purification. Dry solvents were purchased as anhydrous reagents from commercial suppliers. ^{1}H and ^{13}C NMR spectra were recorded on a Bruker Avance DRX AV400 (400 MHz and 101 MHz), AV500 (500 MHz and 125 MHz), AV600 (600 MHz and 151 MHz). ^{1}H chemical shifts are reported in δ (ppm) as s (singlet), d (doublet), dd (doublet of doublet), t (triplet), q (quartet), m (multiplet) and br's (broad singlet) and are referenced to the residual solvent signal: $CDCl_3$ (7.26), DMSO-d_6 (2.50) or MeOD-d_4 (3.34). ^{13}C spectra are referenced to residual solvent signal: $CDCl_3$ (77.1), DMSO-d_6 (39.52) or MeOD-d_4 (49.86). Analytical TLC was carried out on Merck 60 F254 aluminium-backed silica gel plates. Compounds were

purified by column chromatography using VWR silica gel (40 - 63 μm particle size) or flash chromatography on a Biotage Isolera One using Büchi Reveleris Silica Cartridges (4 - 120 g) monitored by UV at λ = 210 nm and 280 nm.

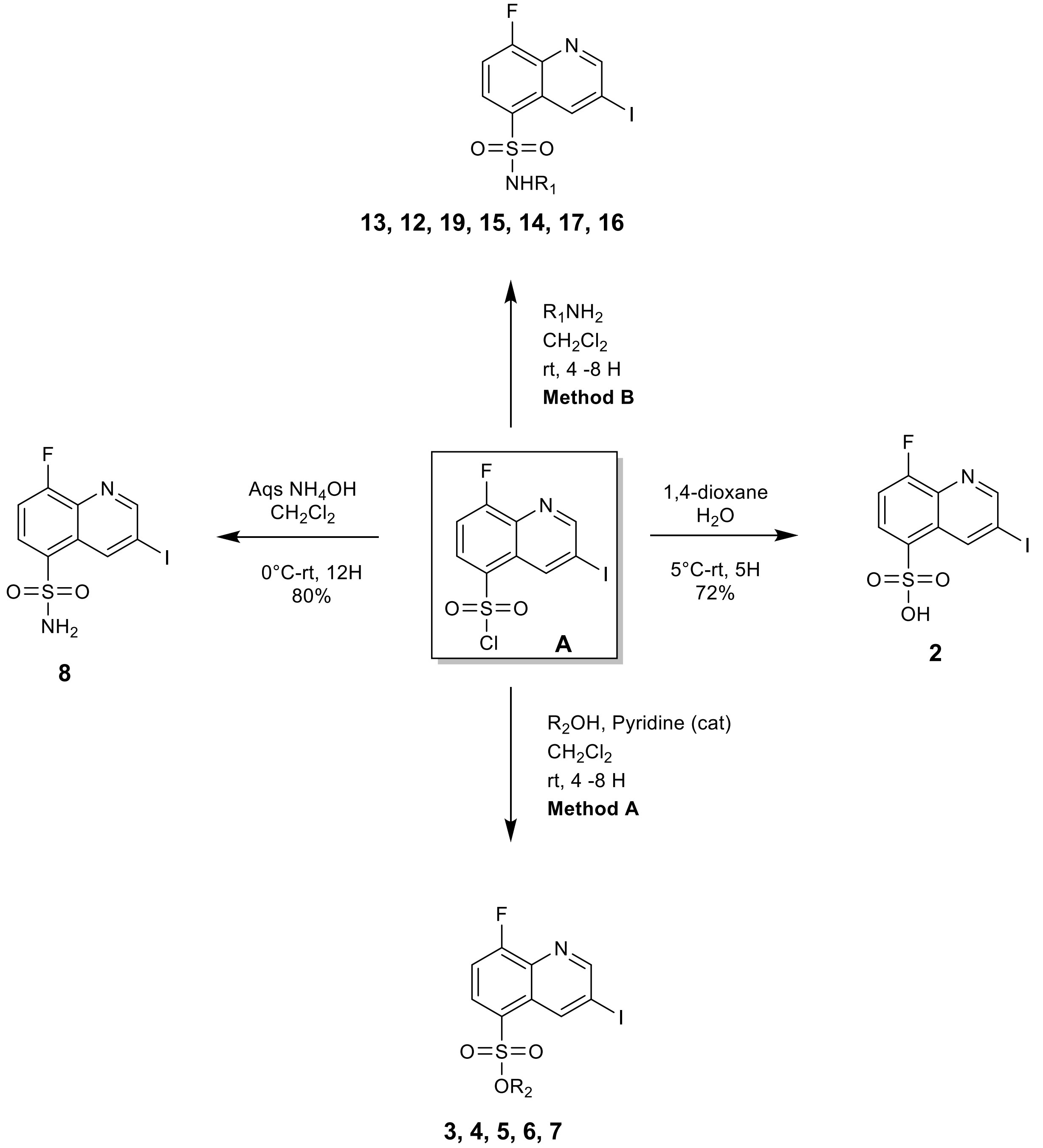

General scheme for the synthesis of sulfonic esters and amides

8-Fluoro-3-iodo-N-(5-methylisoxazol-4-yl)quinoline-5-sulfonamide (1)

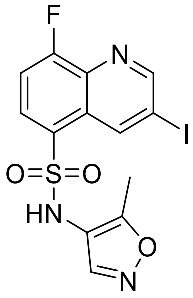

8-Fluoro-3-iodoquinoline-5-sulfonyl chloride (100 µmol, 37 mg) was dissolved in DCM (0.1 M) and 5-methylisoxazol-4-amine hydrochloride (300 µmol, 40 mg) dissolved in pyridine (0.3 M) was added at -10 °C. The reaction mixture was stirred for 10 min before the solution was separated between DCM and bicarb-solution. The aqueous phase was extracted two additional times with DCM and the combined organic phases were dried over Na_2SO_4 and dried in vacuo. The crude product was purified by silica gel flash chromatography using a petrol ether/EtOAc gradient. The product could be obtained as a white solid with a yield of 80% (80 µmol, 35 mg). **^{1}H NMR** (600 MHz, DMSO-*d*6) d ppm 1.87 (s, 3 H) 7.72 (dd, *J*=9.72, 8.62 Hz, 1 H) 8.05 (dd, *J*=8.07, 4.77 Hz, 1 H) 8.26 (s, 1 H) 9.28 (d, *J*=1.83 Hz, 1 H) 9.38 (s, 1 H) 10.33 (br. s., 1 H). **^{13}C NMR** (151 MHz, DMSO-*d*6) d ppm 9.49, 95.69, 112.62, 113.06, 113.19, 126.22, 126.23, 129.35, 129.39, 131.75, 131.82, 136.14, 136.22, 140.29, 149.76, 156.74, 159.60, 161.35, 164.28. **LC-MS** (ESI+) (m/z) calculated for $[C_{13}H_{10}FIN_3O_3S]^+$ 433.95, found 433.95.

8-Fluoro-3-iodo-5-(pyrrolidin-1-ylsulfonyl)quinoline (10)

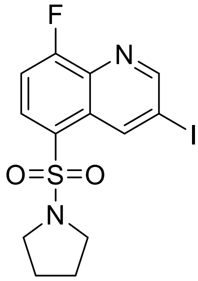

Pyrrolidine (75 µmol, 5.3 mg) was dissolved in DCM (0.1 M) and DIPEA (250 µmol, 32 mg) was added. After 15 min 8-fluoro-3-iodoquinoline-5-sulfonyl chloride (50 µmol, 19 mg) dissolved in DCM (0.1 M) was added at 0 °C. The reaction was stirred for 30 min before the solution was separated between DCM and bicarb-solution. The aqueous phase was extracted two additional times with DCM and the combined organic phases were dried over Na_2SO_4 and dried in vacuo. The crude product was purified by silica gel flash chromatography using a

petrol ether/EtOAc gradient. The product could be obtained as a white solid with a yield of 98% (49 µmol, 20 mg). **^{1}H NMR** (700 MHz, CHLOROFORM-*d*) d ppm 1.84 - 1.92 (m, 4 H) 3.29 - 3.37 (m, 4 H) 7.49 (dd, *J*=9.14, 8.28 Hz, 1 H) 8.24 (dd, *J*=8.28, 4.84 Hz, 1 H) 9.18 (d, *J*=1.94 Hz, 1 H) 9.55 (t, *J*=1.61 Hz, 1 H). **^{13}C NMR** (176 MHz, CHLOROFORM-*d*) d ppm 25.55, 47.58, 93.94, 112.49, 112.60, 127.88, 127.89, 129.40, 129.43, 131.19, 131.25, 136.84, 136.91, 141.63, 141.64, 156.58, 156.59, 160.24, 161.76. **LC-MS** (ESI+) (m/z) calculated for $[C_{13}H_{13}FIN_2O_2S]^+$ 406.97, found 407.00.

***N*^{1}-(3-Iodo-5-(pyrrolidin-1-ylsulfonyl)quinolin-8-yl)-*N*^{1},*N*^{2},*N*^{2}-trimethylethane-1,2-diamine (18)**

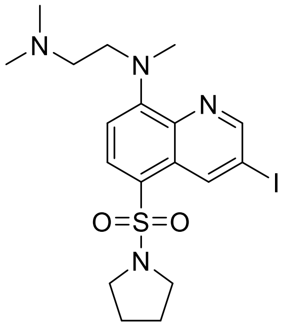

10 (40 µmol, 16 mg) was dissolved in DMF (0.1 M) and *N*^{1},*N*^{1},*N*^{2}-trimethylethane-1,2-diamine (60 µmol, 6.1 mg) and K_2CO_3 (80 µmol, 11 mg) were added. The reaction mixture was stirred at 40 °C for 4 h before the solution was separated between DCM and bicarb-solution. The aqueous phase was extracted two additional times with DCM and the combined organic phases were dried over Na_2SO_4 and dried in vacuo. The crude product was purified by silica gel flash chromatography using a DCM/MeOH gradient. The product could be obtained as an off-white solid with a yield of 85% (34 µmol, 17 mg). **^{1}H NMR** (500 MHz, CHLOROFORM-*d*) d ppm 1.84 (dt, *J*=6.64, 3.55 Hz, 4 H) 2.36 (s, 6 H) 2.84 (t, *J*=7.32 Hz, 2 H) 3.16 (s, 3 H) 3.23 - 3.31 (m, 4 H) 3.93 (t, *J*=7.30 Hz, 2 H) 6.90 (d, *J*=8.85 Hz, 1 H) 8.08 (d, *J*=8.54 Hz, 1 H) 8.93 (d, *J*=2.14 Hz, 1 H) 9.41 (d, *J*=1.83 Hz, 1 H). **^{13}C NMR** (126 MHz, CHLOROFORM-*d*) d ppm 25.47, 40.99, 45.61, 47.32, 54.24, 57.35, 92.49, 110.71, 120.65, 128.24, 132.43, 139.05, 141.41, 151.58, 153.12. **LC-MS** (ESI+) (m/z) calculated for $[C_{18}H_{26}IN_4O_2S]^+$ 489.08, found 488.97.

8-Fluoro-3-iodo-N-phenylquinoline-5-sulfonamide (11)

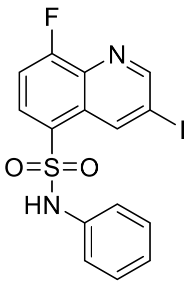

Aniline (75 µmol, 7.0 mg) was dissolved in DCM (0.1 M) and DIPEA (150 µmol, 26 µL) was added. After 15 min 8-fluoro-3-iodoquinoline-5-sulfonyl chloride (50 µmol, 19 mg) dissolved in DCM (0.1 M) was added at 0 °C. The reaction was stirred for 6 h before the solution was separated between DCM and bicarb-solution. The aqueous phase was extracted two additional times with DCM and the combined organic phases were dried over Na_2SO_4 and dried in vacuo. The crude product was purified by silica gel flash chromatography using a petrol ether/EtOAc gradient. The product could be obtained as an off-white solid with a yield of 64% (32 µmol, 14 mg). **^{1}H NMR** (500 MHz, CHLOROFORM-*d*) d ppm 6.82 (s, 1 H) 6.95 - 7.00 (m, 2 H) 7.14 - 7.19 (m, 1 H) 7.21 - 7.26 (m, 2 H) 7.43 (t, *J*=8.70 Hz, 1 H) 8.25 (dd, *J*=8.24, 4.88 Hz, 1 H) 9.14 (d, *J*=1.83 Hz, 1 H) 9.23 (t, *J*=1.68 Hz, 1 H). **^{13}C NMR** (126 MHz, CHLOROFORM-*d*) d ppm 112.32, 112.48, 122.93, 126.58, 126.94, 126.96, 129.31, 129.44, 129.47, 131.56, 131.64, 134.90, 136.32, 136.42, 140.65, 140.66, 156.48, 156.50, 159.95, 162.08. **LC-MS** (ESI+) (m/z) calculated for $[C_{15}H_{11}FIN_2O_2S]^+$ 428.96, found 428.98.

8-fluoro-3-iodoquinoline-5-sulfonic acid (2)

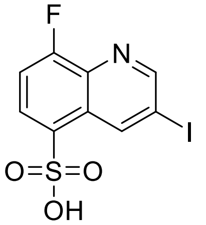

Water (1 mL) was slowly added to the stirred solution of quinoline-5-sulfonyl chloride (**A**) (30 mg, 0.1 mmol) in 1, 4-dioxane (2 mL) at 5 °C and the reaction was allowed to stir for 4-5 hours at room temperature. Excess solvent was evaporated and the crude was purified by CC to afford the title product as off white solid (72%). ^{1}H NMR (500 MHz, $CDCl_3$) δ 9.41 (s, 1H), 9.21 (d, *J* = 1.7 Hz, 1H), 8.37 (dd, *J* = 8.5, 4.6 Hz, 1H), 7.49 (t, *J* = 8.6 Hz, 1H). ^{13}C NMR (126 MHz, $CDCl_3$) δ 162.93 (d, $J_{C\text{-}F}$= 272.1 Hz), 157.69 (s), 140.24 (d, *J* = 1.9 Hz), 136.87 (d, *J* = 12.1 Hz), 134.01 (d, *J* = 4.9 Hz), 131.75 (d, *J* = 10.7 Hz), 126.63 (d, *J* = 2.8

Hz), 112.66 (d, J = 20.9 Hz), 95.81 (s). **LC-MS** (ESI+) m/z calculated for $C_9H_5FINO_3S$ (M + H^+) 353,11, found 353.05.

8-fluoro-3-iodoquinoline-5-sulfonamide (8)

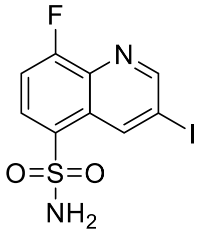

A solution of sat. aqs NH_4OH (1 mL) was added to a cold (0°C) solution of quinoline-5-sulfonyl chloride (**A**) (30 mg, 0.1 mmol) in dioxane (1 mL) and the reaction mixture was allowed to stir for overnight at RT. Water (10 mL) was added and extracted twice with EtOAc (20 mL) and twice with DCM (20 mL). The combined organic fractions were dried over Na_2SO_4 and the solvent was evaporated to afford the title product which was purified by column chromatography provides the title compound as pale yellow solid (80%). ^{1}H NMR (700 MHz, DMSO) δ 9.41 (s, 1H), 9.25 (s, 1H), 8.22 (dd, J = 7.5, 4.7 Hz, 1H), 7.91 (s, 2H), 7.78 (t, J = 9.0 Hz, 1H). ^{13}C NMR (600 MHz, DMSO) δ 160.00 (d, $J_{C\text{-}F}$ = 262.2 Hz), 156.96 (d, J = 6.2 Hz), 141.24 (s), 136.51 (d, J = 12.1 Hz), 135.17 (d, J = 4.6 Hz), 129.29 (d, J = 9.5 Hz), 126.50 (s), 113.40 (d, J = 19.7 Hz), 95.60 (s). **LC-MS** (ESI+) m/z calculated for $C_9H_6FIN_2O_2S$, 352,12, found 353.05 (M + H^+).

7.1 <u>General procedure for the preparation of sulfonic esters (Method A)</u>

Excess equivalents of corresponding alcohol (0.5 mL) was slowly added to the stirred solution of quinoline-5-sulfonyl chloride (30 mg, 0.1 mmol) in dry DCM (2 mL) containing one drop of pyridine (catalytic) at 0 °C in a separate reaction vials and the reaction was allowed to proceed 4-8 hours at RT. Water was added after completion of the reaction and extracted twice with EtOAc (15 mL). The combined organic fractions were dried and the solvent was evaporated. Crude RM was purified by CC to afford the corresponding Quinoline sulfonic ester derivatives.

methyl 8-fluoro-3-iodoquinoline-5-sulfonate (3)

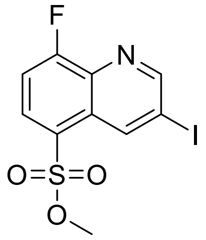

Method A. Pale yellow solid (71%). ^{1}H NMR (400 MHz, $CDCl_3$) δ 9.23 (t, *J* = 1.7 Hz, 1H), 9.16 (d, *J* = 1.9 Hz, 1H), 8.25 (dd, *J* = 8.3, 4.8 Hz, 1H), 7.45 (dd, *J* = 8.9, 8.5 Hz, 1H), 3.71 (s, 3H). ^{13}C NMR (400 MHz, $CDCl_3$) δ 161.02 (d, $J_{C\text{-}F}$ = 269.1 Hz), 156.27 (d, *J* = 1.6 Hz), 140.15 (s), 136.56 – 134.98 (m), 131.51 (d, *J* = 10.0 Hz), 126.62 – 126.29 (m), 124.83 – 124.47 (m), 111.50 (d, *J* = 20.5 Hz), 93.89 (s), 55.84 (s). **LC-MS** (ESI+) m/z calculated for $C_{10}H_7FINO_3S$, 367.13, found 367.97 (M + H^+).

ethyl 8-fluoro-3-iodoquinoline-5-sulfonate (4)

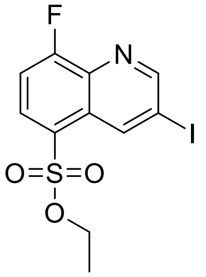

Method A. Pale yellow solid (69%). ^{1}H NMR (600 MHz, DMSO) δ 9.32 (d, *J* = 1.8 Hz, 1H), 9.14 (s, 1H), 8.36 (dd, *J* = 8.3, 4.7 Hz, 1H), 7.86 (dd, *J* = 9.6, 8.5 Hz, 1H), 4.13 (q, *J* = 7.0 Hz, 2H), 1.17 (t, *J* = 7.0 Hz, 3H). ^{13}C NMR (600 MHz, DMSO) δ 161.63 (d, $J_{C\text{-}F}$ = 265.6 Hz), 157.49 (d, *J* = 7.8 Hz), 140.33 (s), 136.73 (d, *J* = 12.4 Hz), 133.36 (d, *J* = 10.6 Hz), 126.95 (d, *J* = 2.3 Hz), 126.23 (d, *J* = 4.6 Hz), 113.89 (d, *J* = 20.4 Hz), 96.66 (s), 69.23 (s), 14.97 (s). **LC-MS** (ESI+) m/z calculated for $C_{11}H_9FINO_3S$, 381.16, found 381.80 (M + H^+).

isopropyl 8-fluoro-3-iodoquinoline-5-sulfonate (5)

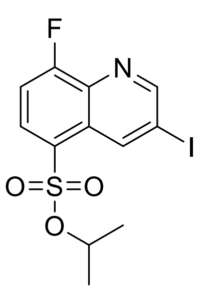

Method A. Pale yellow solid (74%). ^{1}H NMR (500 MHz, $CDCl_3$) δ 9.25 (t, *J* = 1.6 Hz, 1H), 9.15 (d, *J* = 1.8 Hz, 1H), 8.25 (dd, *J* = 8.3, 4.8 Hz, 1H), 7.43 (dd, *J* = 17.7, 8.8 Hz, 1H), 4.78 –

4.69 (m, 1H), 1.20 (d, J = 6.3 Hz, 1H). ^{13}C NMR (500 MHz, $CDCl_3$) δ 161.74 (d, $J_{C\text{-}F}$ = 268.3 Hz), 157.15 (s), 141.35 (d, J = 2.0 Hz), 136.88 (d, J = 12.2 Hz), 131.61 (d, J = 9.9 Hz), 127.97 (d, J = 5.1 Hz), 127.35 (s), 112.57 (d, J = 20.4 Hz), 94.52 (s), 78.80 (s), 22.81 (s). **LC-MS** (ESI+) m/z calculated for $C_{12}H_{11}FINO_3S$, 395.19, found 395.90.

pentyl 8-fluoro-3-iodoquinoline-5-sulfonate (6)

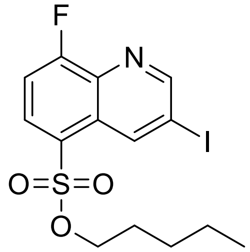

Method A. Off white solid (76%). ^{1}H NMR (700 MHz, $CDCl_3$) δ 9.26 (t, J = 1.5 Hz, 1H), 9.15 (d, J = 1.9 Hz, 1H), 8.24 (dd, J = 8.3, 4.7 Hz, 1H), 7.44 (t, J = 8.7 Hz, 1H), 3.99 (t, J = 6.5 Hz, 2H), 1.61 – 1.54 (m, 3H), 1.18 – 1.10 (m, 5H), 0.75 (t, J = 7.1 Hz, 3H). ^{13}C NMR (700 MHz, $CDCl_3$) δ 160.85 (d, $J_{C\text{-}F}$ = 268.6 Hz), 156.18 (s), 140.24 (d, J = 1.9 Hz), 135.89 (d, J = 12.1 Hz), 131.09 (d, J = 9.9 Hz), 126.38 (d, J = 2.4 Hz), 125.76 (d, J = 5.0 Hz), 111.51 (d, J = 20.3 Hz), 93.61 (s), 70.69 (s), 27.40 (s), 26.51 (s), 20.92 (s), 12.79 (s). **LC-MS** (ESI+) m/z calculated for $C_{14}H_{15}FINO_3S$, 423.24, found 423.76.

General scheme for the synthesis of reverse sulphonamides (Sun et al., 2015):

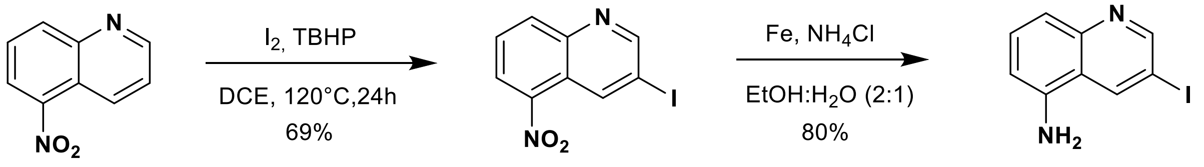

3-iodoquinolin-5-amine:

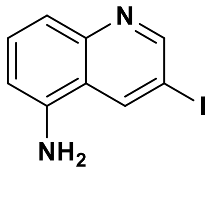

Yellow solid (80%). 3-iodo-5-nitroquinoline (1.4 g, 5 mmol), Iron powder (782 mg, 15 mmol) and NH_4Cl (1.25 g, 25 mmol) were dissolved in EtOH:H_2O (21 mL, 2:1 ratio) in a 50 mL RB flask and allowed to stir for 12 h at 60°C. After completion of the reaction monitored by TLC, filtered through a pad of celite and wash with EtOAc (25 mL). The filtrate was

concentrate and crude mixture was purified by CC. ^{1}H NMR (500 MHz, $CDCl_3$) δ 8.88 (d, *J* = 2.0 Hz, 1H), 8.50 (d, *J* = 1.4 Hz, 1H), 7.43 (dt, *J* = 13.6, 8.3 Hz, 2H), 6.75 (dd, *J* = 7.1, 1.2 Hz, 1H). ^{13}C NMR (500 MHz, $CDCl_3$) δ 155.10 (s), 146.82 (s), 141.56 (s), 138.19 (s), 130.84 (s), 120.53 (s), 119.53 (s), 110.94 (s), 87.46 (s). **LC-MS** (ESI+) m/z calculated for $C_9H_7IN_2$, 270.07, found 270.46.

N-(3-iodoquinolin-5-yl)-4-methoxybenzenesulfonamide (19)

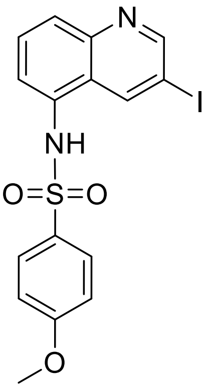

4-Methoxy Sulfonyl chloride (**A**) (25 mg, 0.1 mmol) was slowly added to the stirred mixture of 3-Iodo-5-amino-Quinoline (30 mg, 0.1 mmol) in dry CH_2Cl_2 (2 mL) at 10 °C and allowed to stir for 12 H at room temperature. After completion of the reaction, monitored by TLC, Crude was purified by CC using EtOAC : Petroleum ether. Off white solid (78%). ^{1}H NMR (500 MHz, DMSO) δ 10.24 (s, 1H), 8.98 (d, *J* = 2.0 Hz, 1H), 8.60 (d, *J* = 1.3 Hz, 1H), 7.86 (d, *J* = 8.5 Hz, 1H), 7.77 – 7.66 (m, 1H), 7.59 – 7.49 (m, 1H), 7.30 (d, *J* = 7.5 Hz, 1H), 7.01 (dd, *J* = 9.4, 2.4 Hz, 1H), 3.80 (s, 1H). ^{13}C NMR (500 MHz, DMSO) δ 163.03 (s), 155.89 (s), 146.63 (s), 139.81 (s), 132.46 (s), 131.22 (s), 130.25 (s), 129.36 (s), 128.01 (s), 126.30 (s), 125.17 (s), 114.85 (s), 91.55 (s), 56.14 (s). **LC-MS** (ESI+) m/z calculated for $C_{16}H_{13}IN_2O_3S$, 440.26, found 440.90.

4-methoxy-N-(3-phenylquinolin-5-yl)benzenesulfonamide (20)

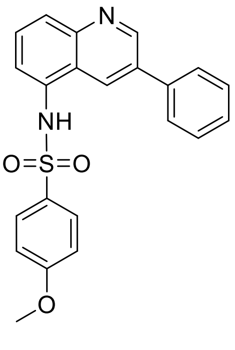

N-(3-iodoquinolin-5-yl)-4-methoxybenzenesulfonamide (30 mg, 0.1 mmol), phenylboronic acid (12.5 mg, 0.1 mmol) and K_2CO_3 (19 mg, 0.1 mmol) were dissolved in 1,4-dioxane (2 mL) and degasify with Argon for 10 min in a small MW vial. $Pd(PPh_3)_4$ (8 mg, 0,01 mmol) was added to the above reaction mixture and allowed to stir for 2 hours at 120 °C under microwave irradiation. After completion of the reaction, monitored by TLC, concentrate the excess solvent and the crude was purified by CC using EtOAC : Petroleum ether. Pale yellow solid (78%). ^{1}H NMR (400 MHz, $CDCl_3$) δ 9.08 (s, 1H), 8.50 (s, 1H), 8.03 (d, *J* = 8.4 Hz, 1H), 7.54 (dt, *J* = 16.0, 8.5 Hz, 6H), 7.44 (t, *J* = 7.4 Hz, 2H), 7.41 – 7.34 (m, 1H), 7.22 (d, *J* = 7.4 Hz, 1H), 6.75 (d, *J* = 8.9 Hz, 2H), 3.68 (s, 3H). ^{13}C NMR (400 MHz, $CDCl_3$) δ 162.28 (s), 136.01 – 135.71 (m), 133.35 – 132.97 (m), 131.05 (s), 129.18 (s), 128.58 (s), 128.25 (s), 127.57 (s), 126.47 (s), 124.39 (s), 113.21 (s), 54.55 (s). **LC-MS** (ESI+) m/z calculated for $C_{22}H_{18}N_2O_3S$, 390.46, found 391.1 (M + H^+).

N-(3-iodoquinolin-5-yl)-4-methylbenzamide (21)

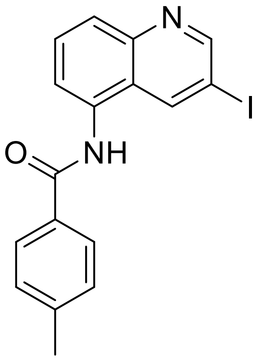

3-Iodo-5-amino-Quinoline (50 mg, 0,18 mmol) ,4-Methylbenzoicacid (30 mg, 0.22 mmol), HATU (74 mg, 0.2 mmol) and DIPEA (0.1 mL, 0.55 mmol) were dissolved in DMF (2 mL) and RM was allowed to stir for 12 H. After completion of the reaction, monitored by TLC, water (2 mL) was added and solids were filtered and washed with water (1 mL). Crude solids were purified by CC using EtOAC : Petroleum ether. Off white solid (84%). ^{1}H NMR (400 MHz, MeOD) δ 8.92 (d, *J* = 2.0 Hz, 1H), 8.65 (dd, *J* = 1.9, 0.7 Hz, 1H), 7.86 (d, *J* = 8.2 Hz, 3H), 7.76 – 7.68 (m, 1H), 7.61 (dd, *J* = 7.4, 0.9 Hz, 1H), 7.28 (d, *J* = 8.0 Hz, 2H), 2.36 (s, 3H). ^{13}C NMR (400 MHz, MeOD) δ 168.53 (s), 155.42 (s), 146.14 (s), 142.89 (s), 140.56 (s), 132.93 (s), 130.84 (s), 129.82 (s), 129.07 (s), 127.64 (s), 126.90 (s), 126.18 (s), 125.01 (s), 89.06 (s), 20.40 (s). **LC-MS** (ESI+) m/z calculated for $C_{17}H_{13}IN_2O$, 388.21, found 389.07 (M + H^+).

7.2 General procedure for the preparation of sulphonamide derivatives (Method B)

Corresponding amines (1.2 eq) were slowly added to the stirred solution of quinoline-5-sulfonyl chloride (**A)** (30 mg, 0.1 mmol) and DIPEA (1.0 eq) in dry DCM (2 mL) at 10 °C in a separate reaction vials and the reaction mixture was allowed to stir for 4-8 hours at RT. Water was added after completion of the reaction and extracted twice with EtOAc (10 mL). The combined organic fractions were dried and the solvent was evaporated. Crude RM was purified by CC using EtOAc: Petroleum ether to afford the corresponding Quinoline sulfonamide derivatives.

N-benzyl-8-fluoro-3-iodoquinoline-5-sulfonamide (13)

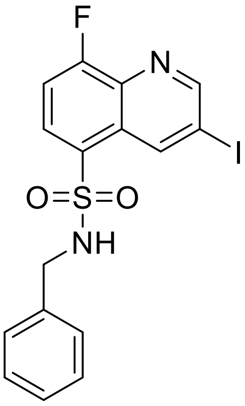

Method B. Pale yellow solid (81%). ^{1}H NMR (600 MHz, $CDCl_3$) δ 9.30 (s, 1H), 9.10 (d, *J* = 1.7 Hz, 1H), 8.16 (dd, J = 8.3, 4.8 Hz, 1H), 7.33 (t, *J* = 8.7 Hz, 1H), 7.14 – 7.04 (m, 3H), 7.00 – 6.92 (m, 2H), 5.04 (t, *J* = 5.7 Hz, 1H), 4.10 (d, *J* = 5.9 Hz, 2H). ^{13}C NMR (600 MHz, $CDCl_3$) δ 160.20 (d, $J_{C\text{-}F}$ = 267.3 Hz), 155.60 (d, *J* = 7.8 Hz), 139.82 (t, *J* = 3.5 Hz), 135.77 (dd, *J* = 17.1, 11.1 Hz), 134.33 (d, *J* = 7.7 Hz), 130.62 (t, *J* = 12.5 Hz), 129.10 (t, *J* = 5.6 Hz), 127.55 (d, *J* = 9.3 Hz), 127.03 (d, *J* = 10.9 Hz), 126.72 (d, *J* = 8.2 Hz), 125.98 (t, *J* = 9.7 Hz), 111.36 (dd, *J* = 29.3, 17.1 Hz), 92.91 (d, *J* = 82.5 Hz), 46.63 – 45.80 (m). **LC-MS** (ESI+) m/z calculated for $C_{16}H_{12}FIN_2O_2S$, 442.25, found 442.93.

N-cyclohexyl-8-fluoro-3-iodoquinoline-5-sulfonamide (12)

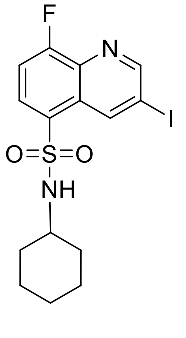

Method B. Off white solid (78%). ^{1}H NMR (500 MHz, $CDCl_3$) δ 9.33 (t, J = 1.5 Hz, 1H), 9.12 (d, J = 1.8 Hz, 1H), 8.25 (dd, J = 8.3, 4.9 Hz, 1H), 7.51 – 7.30 (m, 1H), 4.61 (d, J = 8.0 Hz, 1H), 3.11 (tdt, J = 11.8, 7.9, 3.9 Hz, 1H), 1.71 – 1.38 (m, 5H), 1.27 – 0.95 (m, 5H). ^{13}C NMR (500 MHz, $CDCl_3$) δ 160.07 (d, $J_{C\text{-}F}$ = 266.7 Hz), 155.68 (s), 139.95 (d, J = 2.0 Hz), 135.91 (d, J = 12.1 Hz), 130.08 (d, J = 9.5 Hz), 125.99 (d, J = 2.1 Hz), 111.52 (d, J = 20.0 Hz), 93.07 (s), 52.08 (s), 33.03 (s), 23.95 (s), 23.63 (s). **LC-MS** (ESI+) m/z calculated for $C_{15}H_{16}FIN_2O_2S$, 434.27, found 434.95.

8-fluoro-3-iodo-N-(2-morpholinoethyl)quinoline-5-sulfonamide (15)

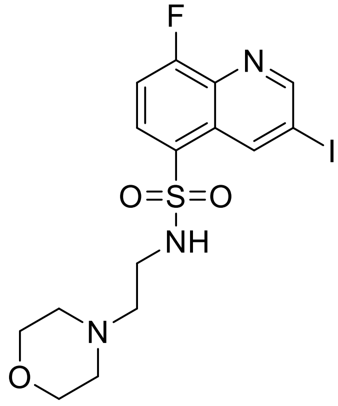

Method B. Pale yellow solid (83%). ^{1}H NMR (400 MHz, $CDCl_3$) δ 9.35 (t, J = 1.7 Hz, 1H), 9.13 (d, J = 1.9 Hz, 1H), 8.23 (dd, J = 8.3, 4.9 Hz, 1H), 7.42 (dd, J = 9.0, 8.4 Hz, 1H), 5.55 (s, 1H), 3.54 – 3.47 (m, 4H), 2.95 – 2.89 (m, 2H), 2.34 – 2.26 (m, 2H), 2.22 – 2.15 (m, 4H). ^{13}C NMR (400 MHz, $CDCl_3$) δ 160.22 (d, $J_{C\text{-}F}$ = 267.3 Hz), 155.77 (s), 139.77 (s), 136.04 (s), 130.64 (d, J = 9.6 Hz), 128.58 (d, J = 5.1 Hz), 126.03 (s), 111.50 (d, J = 20.0 Hz), 93.30 (s), 65.58 (s), 55.09 (s), 51.94 (s), 37.81 (s). **LC-MS** (ESI+) m/z calculated for $C_{15}H_{17}FIN_3O_3S$, 465.28, found 466.04 ($M + H^+$).

8-fluoro-3-iodo-N-(3-methoxyphenyl)quinoline-5-sulfonamide (17)

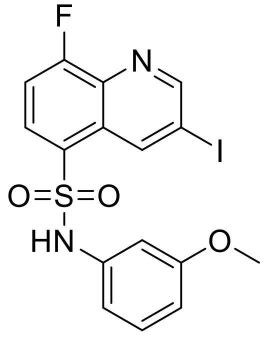

Method B. Pale yellow solid (80%). ^{1}H NMR (600 MHz, $CDCl_3$) δ 9.21 (t, *J* = 1.4 Hz, 1H), 9.06 (d, *J* = 1.8 Hz, 1H), 8.20 (dd, *J* = 8.4, 4.8 Hz, 1H), 7.35 (t, *J* = 8.7 Hz, 1H), 7.19 (d, *J* = 9.0 Hz, 1H), 7.01 (t, *J* = 8.1 Hz, 1H), 6.61 – 6.58 (m, 1H), 6.56 (t, *J* = 2.2 Hz, 1H), 6.42 (dd, *J* = 7.9, 1.4 Hz, 1H), 3.65 (s, 3H). ^{13}C NMR (600 MHz, $CDCl_3$) δ 160.26 (d, $J_{C\text{-}F}$ = 267.7 Hz), 159.36 (s), 155.66 (d, *J* = 20.3 Hz), 139.89 (t, *J* = 22.3 Hz), 135.63 (d, *J* = 12.0 Hz), 135.55 (s), 130.85 (d, *J* = 9.8 Hz), 129.22 (s), 128.82 (d, *J* = 5.0 Hz), 126.26 (d, *J* = 2.0 Hz), 113.61 (s), 111.65 (d, *J* = 20.1 Hz), 110.88 (s), 107.62 (s), 93.23 (s), 54.35 (s). MS (ESI). **LC-MS** (ESI+) m/z calculated for $C_{16}H_{12}FIN_2O_3S$, 458.25, found 458.96.

8-fluoro-3-iodo-N-phenethylquinoline-5-sulfonamide (14)

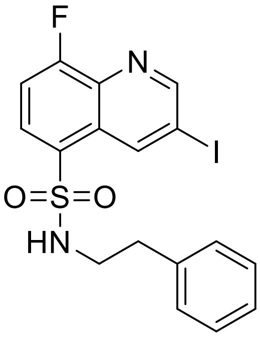

Method B. Pale yellow solid (79%). ^{1}H NMR (400 MHz, $CDCl_3$) δ 9.08 (dt, *J* = 4.0, 1.9 Hz, 1H), 8.15 (dd, *J* = 8.3, 4.9 Hz, 1H), 7.37 (dd, *J* = 9.1, 8.4 Hz, 1H), 7.08 – 6.95 (m, 2H), 6.86 – 6.73 (m, 1H), 4.57 (t, *J* = 6.0 Hz, 1H), 3.18 (dd, *J* = 12.8, 6.4 Hz, 1H), 2.62 (t, *J* = 6.5 Hz, 1H). ^{13}C NMR (400 MHz, $CDCl_3$) δ 161.27 (d, $J_{C\text{-}F}$ = 266.9 Hz), 156.62 (s), 140.76 (d, *J* = 2.1 Hz), 136.98 (s), 136.97 – 136.94 (m), 136.84 (s), 131.53 (d, *J* = 9.7 Hz), 129.62 (d, *J* = 5.0 Hz), 128.63 (s), 128.34 (s), 126.96 (d, *J* = 2.3 Hz), 126.92 (s), 112.32 (d, *J* = 20.1 Hz), 94.35 (s), 44.18 (s), 35.43 (s). **LC-MS** (ESI+) m/z calculated for $C_{17}H_{14}FIN_2O_2S$, 456.27, found 457.08 ($M + H^+$).

3-aminophenyl 8-fluoro-3-iodoquinoline-5-sulfonate (7)

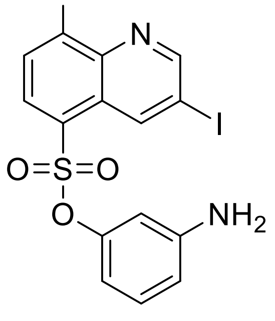

Method A. Off white solid (84%). ^{1}H NMR (600 MHz, MeOD) δ 9.46 (t, J = 1.5 Hz, 1H), 9.12 (d, J = 1.9 Hz, 1H), 8.29 (dd, J = 8.4, 4.8 Hz, 1H), 7.56 (dd, J = 9.5, 8.5 Hz, 1H), 6.96 (t, J = 8.1 Hz, 1H), 6.52 (t, J = 2.2 Hz, 1H), 6.48 (ddd, J = 8.2, 2.3, 0.8 Hz, 1H), 6.42 (ddd, J = 8.0, 2.0, 0.8 Hz, 1H). ^{13}C NMR (600 MHz, MeOD) δ 160.68 (d, $J_{C\text{-}F}$ = 264.9 Hz), 157.94 (s), 156.43 (s), 141.47 (d, J = 1.6 Hz), 137.68 (s), 136.08 (d, J = 12.2 Hz), 131.93 (d, J = 9.7 Hz), 130.47 (d, J = 4.9 Hz), 129.60 (s), 126.93 (d, J = 1.7 Hz), 112.39 (t, J = 10.1 Hz), 112.13 (s), 108.42 (s), 93.18 (s). **LC-MS** (ESI+) m/z calculated for $C_{15}H_{10}FIN_2O_3S$, 444.22, found 445.0 ($M+H^+$).

8-fluoro-N-(3-hydroxyphenyl)-3-iodoquinoline-5-sulfonamide (16)

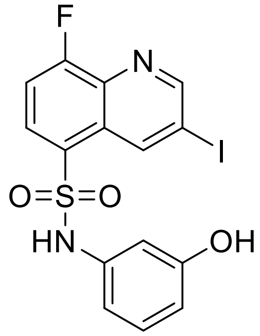

N-(3-((tert-butyldimethylsilyl)oxy)phenyl)-8-fluoro-3-iodoquinoline-5-sulfonamide was prepared by using **Method B** with readily available sulphonyl chloride **A** and 3-((tert-butyldimethylsilyl)oxy)aniline. TBS group was deprotected by stirring the compound (100 mg, 0.18 mmol) with K_2CO_3 (13 mg, 0.09 eq) in 2 mL DMF:H_2O (10:1) mixture at room temperature for 6 hours. Quench by adding water (5 mL) and solids were filtered and washed with water and dried. Crude was purified by CC using EtOAc : Pertroleum ether to afford the title product as pale yellow solid (66% overall two steps). ^{1}H NMR (600 MHz, DMSO) δ 10.71 (s, 1H), 9.45 (s, 1H), 9.44 (s, 1H), 9.24 (d, J = 1.6 Hz, 1H), 8.23 (dd, J = 8.3, 4.7 Hz, 1H), 7.75 (t, J = 9.0 Hz, 1H), 6.95 (t, J = 8.0 Hz, 1H), 6.44 (d, J = 8.1 Hz, 2H), 6.40 – 6.36 (m, 1H). ^{13}C NMR (600 MHz, DMSO) δ 160.70 (d, $J_{C\text{-}F}$ = 264.0 Hz), 158.38 (s), 157.22 (s), 140.57 (s), 138.26 (s), 136.42 (d, J = 12.0 Hz), 132.49 (d, J = 9.9 Hz), 130.39 (d, J = 17.2 Hz), 130.29 (d, J = 4.4 Hz), 126.69 (s), 113.44 (d, J = 19.9 Hz), 112.09 (s), 111.09 (s), 107.53 (s), 96.23 (s). **LC-MS** (ESI+) m/z calculated for $C_{15}H_{10}FIN_2O_3S$, 444.22, found 444.94.

8-fluoro-3-iodo-N-methylquinoline-5-sulfonamide (9)

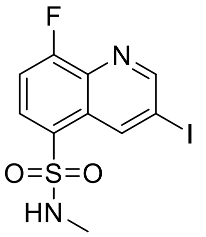

Method B. Pale yellow solid. ^{1}H NMR (500 MHz, $CDCl_3$) δ 9.34 (t, *J* = 1.7 Hz, 1H), 9.13 (d, *J* = 1.9 Hz, 1H), 8.23 (dd, *J* = 8.3, 4.9 Hz, 1H), 7.42 (dt, *J* = 11.1, 5.6 Hz, 1H), 4.67 (d, *J* = 5.2 Hz, 1H), 2.59 (d, *J* = 5.3 Hz, 3H). ^{13}C NMR (500 MHz, $CDCl_3$) δ 161.30 (d, $J_{C\text{-}F}$ = 267.0 Hz), 156.81 (s), 140.98 (s), 137.09 (s), 131.91 (d, *J* = 9.7 Hz), 128.98 (d, *J* = 5.2 Hz), 127.21 (s), 112.50 (d, *J* = 20.1 Hz), 94.39 (s), 29.28 (s). **LC-MS** (ESI+) m/z calculated for $C_{10}H_8FIN_2O_2S$, 366.15, found 366.94.

9. LC-MS analysis of the compounds:

High-resolution electrospray ionization mass spectra (ESI-FTMS) were recorded on a Thermo LTQ Orbitrap (high-resolution mass spectrometer) coupled to an Accela HPLC system supplied with a Hypersil GOLD column (Thermo Electron). LCMS (ESI-MS) analysis was performed using Agilent HPLC system (1100 series) with CC 125/4 Nucleodur C18 gravity column (3 µm) from Macherey Nagel coupled to a Thermo Scientific Finnigan LCQ Advantage Max Ion Trap and ESA Corona detector. HPLC was recorded at a flow rate of 1 mL/min in a 15 min run comprising of initial 1 min water:ACN (90:10), increasing gradient for 10 min to 100% ACN, 2 min hold at 100% ACN, then back to water:ACN (90:10) in 0.5 min, plus 1.5 min hold at water:ACN (90:10) to reach the starting conditions.

10. Molecular docking:

The protein structure (PDB-ID 5deu) was prepared with Protoss (Bietz et al., 2014) as implemented the Proteins*Plus* web server(Schöning-Stierand et al., 2020). The small molecules were prepared for docking using UNICON (Sommer et al., 2016) retaining only the most probable protonation states and generating initial three-dimensional conformations. Apart from this, default settings were used. We applied the molecular docking software GOLD (Jones et al., 1997) to predict potential binding modes of the small molecules in the 5mC (ligand ID of the reference ligand: 5HC) and the α-KG (ligand ID of the reference ligand: OGA) binding site. All non-protein residues were removed except for HOH 2113 which is involved in metal coordination. For this water molecule, translation by 0.5 Å,

toggling and spinning was enabled. Automated binding site detection was disabled, and the binding site was restricted to all atoms in a 10 Å environment of the reference ligand atoms. Per ligand, 100 GA runs were performed with a search efficiency of 2. We performed two docking runs for the docking with α-KG as reference ligand: a) the Fe^{2+} ion was retained as metal ion in the binding site 2) the Fe^{2+} ion was removed from the binding site. Apart from this, default settings were applied. The docking results of all docking runs were visually inspected. For most molecules, the three highest scored poses were highly similar. These poses were chosen for the investigation of potential binding modes of the hit compound **2**.

11. Binding Site Comparison:

The preparation of the binding sites from the scPDB (Meslamani et al., 2011) was performed as described elsewhere(Ehrt et al., 2018). The same preparation steps were applied to the structure of TET2 (PDB-ID 5deu). The results of the comparison of the 2-oxogluatarate binding site of TET2 with all binding sites in the scPD with IsoMIF (Chartier et al., 2016) can be found in the SI. The IsoMIF-based alignment of the structure of HIF-1 (PDB-ID 3od4) was used to align FTO (PDB-ID 4ie4) to TET2.

12. DFT Calculations:

The complex model shown in Fig. S15 was geometry-optimized on ωB97X-D/dev2-SVP level of theory using the Wavefunction Spartan '18 software package and obtained structures subjected to ωB97X-D/dev2-TZVP single point energy calculations. To keep the electronic situation of the transition metal center as simple as possible and comparable for all herein compared coordination environments, comprising different ligand combinations, the even-electron-count d^6 octahedral Fe(II) center was treated as a low-spin complex (spin multiplicity = 1) using restricted wave function computations for all examined complexes, even though spectroscopic studies on related water-substituted (but alpha-ketoglutarate-unbound) Fe(II) complexes in the resting states of alpha-ketoglutarate-dependent oxidases with one carboxylate (or halide) and two histidine ligands indicated a high-spin (S = 2) situation (Chang, 2018). In the following, the deprotonated 8HQ ligand was replaced by the 8-fluoroquinoline ligand motif found in compound **2**, further by 8HQ (non-deprotonated), quinoline and pyridine, to learn about the relative energies leading to formation of these coordination environments by ligand exchange. Therefore, complex formation reactions shown in Fig. S16 were computed by performing unconstrained gas-phase geometry optimizations on DFT ωB97X-D/dev2-SVP level for all components, followed by ωB97X-D/dev2-TZVP single point calculations.

APPENDIX

1. Plasmid map

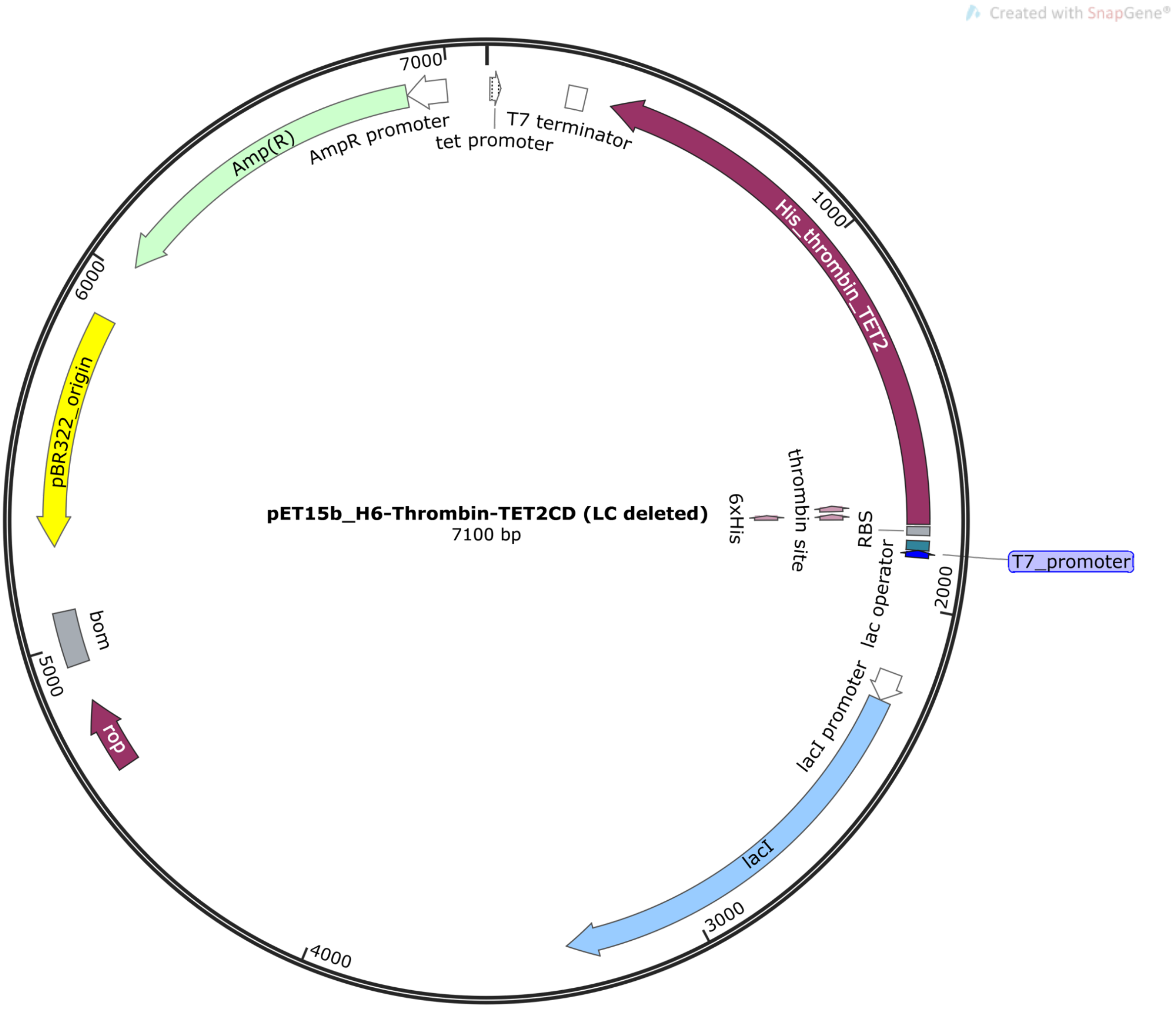

Plasmid map of hTET2 catalytic domain (CD) used in the study (Low complexity insert deleted).

Supplementary References:

Bietz, S., Urbaczek, S., Schulz, B., and Rarey, M. (2014). Protoss: a holistic approach to predict tautomers and protonation states in protein-ligand complexes. J Cheminform *6*, 12. 10.1186/1758-2946-6-12.

Chang, W., Liu, P., and Guo, Y. (2018) Mechanistic Elucidation of Two Catalytically Versatile Iron(II)- and α-Ketoglutarate-Dependent Enzymes: Cases Beyond Hydroxylation. Comments on Inorganic Chemistry, *38*, 127-165. 10.1080/02603594.2018.1509856

Chartier, M., Adriansen, E., and Najmanovich, R. (2016). IsoMIF Finder: online detection of binding site molecular interaction field similarities. Bioinformatics *32*, 621-623. 10.1093/bioinformatics/btv616.

Ehrt, C., Brinkjost, T., and Koch, O. (2018). A benchmark driven guide to binding site comparison: An exhaustive evaluation using tailor-made data sets (ProSPECCTs). PLOS Computational Biology *14*, e1006483. 10.1371/journal.pcbi.1006483.

Gibb, S. (2019). MALDIquantForeign: Import/Export Routines for 'MALDIquant'. R package version 0.12. https://cran.r-project.org/web/packages/MALDIquantForeign/index.html.

Gibb, S., and Strimmer, K. (2012). MALDIquant: a versatile R package for the analysis of mass spectrometry data. Bioinformatics *28*, 2270-2271. 10.1093/bioinformatics/bts447.

Hu, L., Li, Z., Cheng, J., Rao, Q., Gong, W., Liu, M., Shi, Y.G., Zhu, J., Wang, P., and Xu, Y. (2013). Crystal structure of TET2-DNA complex: insight into TET-mediated 5mC oxidation. Cell *155*, 1545-1555. 10.1016/j.cell.2013.11.020.

Jones, G., Willett, P., Glen, R.C., Leach, A.R., and Taylor, R. (1997). Development and validation of a genetic algorithm for flexible docking. J Mol Biol *267*, 727-748. 10.1006/jmbi.1996.0897.

Meslamani, J., Rognan, D., and Kellenberger, E. (2011). sc-PDB: a database for identifying variations and multiplicity of 'druggable' binding sites in proteins. Bioinformatics *27*, 1324-1326. 10.1093/bioinformatics/btr120.

Pettersen, E.F., Goddard, T.D., Huang, C.C., Couch, G.S., Greenblatt, D.M., Meng, E.C., and Ferrin, T.E. (2004). UCSF Chimera--a visualization system for exploratory research and analysis. J Comput Chem *25*, 1605-1612. 10.1002/jcc.20084.

R: A language and environment for statistical computing. (2021). https://www.R-project.org/.

Ritz, C., Baty, F., Streibig, J.C., and Gerhard, D. (2016). Dose-Response Analysis Using R. PLOS ONE *10*, e0146021. 10.1371/journal.pone.0146021.

Sappa, S., Dey, D., Sudhamalla, B., and Islam, K. (2021). Catalytic Space Engineering as a Strategy to Activate C-H Oxidation on 5-Methylcytosine in Mammalian Genome. J Am Chem Soc *143*, 11891-11896. 10.1021/jacs.1c03815.

Schöning-Stierand, K., Diedrich, K., Fährrolfes, R., Flachsenberg, F., Meyder, A., Nittinger, E., Steinegger, R., and Rarey, M. (2020). ProteinsPlus: interactive analysis of protein-ligand binding interfaces. Nucleic Acids Res *48*, W48-w53. 10.1093/nar/gkaa235.

Sommer, K., Friedrich, N.O., Bietz, S., Hilbig, M., Inhester, T., and Rarey, M. (2016). UNICON: A Powerful and Easy-to-Use Compound Library Converter. J Chem Inf Model *56*, 1105-1111. 10.1021/acs.jcim.6b00069.

Sun, K., Lv, Y., Wang, J., Sun, J., Liu, L., Jia, M., Liu, X., Li, Z., and Wang, X. (2015). Regioselective, Molecular Iodine-Mediated C3 Iodination of Quinolines. Org Lett *17*, 4408-4411. 10.1021/acs.orglett.5b01857.

Wickham, H. (2019). Welcome to the tidyverse. Journal of Open Source Software *4*.

Wolle, P., Weisner, J., Keul, M., Landel, I., Lategahn, J., and Rauh, D. (2018). RASPELD to Perform High-End Screening in an Academic Environment toward the Development of Cancer Therapeutics. ChemMedChem *13*, 2065-2072. 10.1002/cmdc.201800477.

Zhang, J.H., Chung, T.D., and Oldenburg, K.R. (1999). A Simple Statistical Parameter for Use in Evaluation and Validation of High Throughput Screening Assays. J Biomol Screen *4*, 67-73. 10.1177/108705719900400206.

Zhang, Z., and Marshall, A.G. (1998). A universal algorithm for fast and automated charge state deconvolution of electrospray mass-to-charge ratio spectra. J Am Soc Mass Spectrom *9*, 225-233. 10.1016/s1044-0305(97)00284-5.
